# Systematic Review of Financing Functions for Universal Health Coverage in Low- and Middle-Income Countries: Reforms, Challenges, and Lessons Learned

**DOI:** 10.3389/phrs.2025.1607745

**Published:** 2025-09-23

**Authors:** Othmane Hajji, Bouchra El Abbadi, El Houcine Akhnif

**Affiliations:** ^1^ Laboratory for Research in Management Transformation and Innovation(LA-TIM), National School of Business and Management, Hassan Premier University, Settat, Morocco; ^2^ World Health Organization Country Office of Morocco, Rabat, Morocco

**Keywords:** health financing functions, fundraising, pooling, purchasing of health services, universal health coverage

## Abstract

**Objective:**

The systematic review reveals a lack of research on financing universal health coverage (UHC) in low- and middle-income countries (LMICs). This study aims to examine the financing mechanisms used, identify the main challenges faced, and gather insights from successful experiences to inform future reforms in LMICs.

**Methods:**

We conducted a literature search across seven academic databases, limiting our systematic review to studies published in English and French between 2010 and 2022, which were then included in our qualitative analysis.

**Results:**

A total of 45 studies met the inclusion criteria—most used qualitative (n = 23) or documentary (n = 15) approaches. The majority (n = 37) were published between 2015 and 2022. Using Kutzin’s framework, we analyzed health financing functions in LMICs. Key challenges and lessons learned were summarized to improve understanding of ongoing financing issues and opportunities for reform.

**Conclusion:**

This study emphasizes key financing strategies and ongoing challenges in LMICs and provides specific recommendations for countries to prioritize reforms and address health financing gaps. The goal is to speed up progress toward UHC.

## Introduction

Since 2005, when the World Health Organization (WHO) member states first adopted a resolution linking the concept of universal health coverage (UHC) to sustainable healthcare financing, global attention to health financing has grown significantly [1]. Several countries have joined this international movement to ensure their populations have equitable access to healthcare services without making their participation in healthcare financing regressive [2].

Although UHC became a global priority in 2010, many low- and middle-income countries (LMICs) have since made significant progress in developing financing systems to support this goal. Countries such as Thailand, Rwanda, and Costa Rica have demonstrated that progress toward UHC can be achieved through diverse models tailored to their specific national contexts [3]. Nevertheless, as the WHO highlighted in 2010, the majority of LMICs were still far from achieving UHC goals at that time [3]. UHC has subsequently received worldwide support for its importance and influence on other social determinants, such as poverty, social inclusion, and employment [4]. This is why the United Nations General Assembly adopted health as one of the Sustainable Development Goals (SDGs) and UHC as a health-related target under the SDGs [5]. However, the question that has been raised since 2010 is how health systems finance this major social project [3].

According to the latest reports published by the WHO and the World Bank, 1.4 to 1.9 billion people face catastrophic or impoverishing health expenses, most of whom are from LMICs [6, 7]. This critical situation is mainly caused by the rapid rise in out-of-pocket healthcare costs, which have surpassed other household expenses. Several factors explain this trend, including the inadequacy of financial protection mechanisms. COVID-19 has only worsened this situation by increasing the number of people skipping care and the financial strain of healthcare costs, especially for low-income populations worldwide [6]. Therefore, adopting an equitable healthcare financing model remains a significant challenge for achieving universal healthcare coverage [8].

Health financing plays a crucial role in any healthcare system, ensuring health equity and fully addressing the health needs of all citizens through its three interconnected functions: raising funds from various sources, pooling resources via prepayment systems, and purchasing healthcare services [9–11].

Health financing models in LMICs tend to rely on funding mechanisms that combine regressive sources (such as direct payments and external sources) at a high rate, with other progressive but limited methods, like tax revenues and contributions from social insurance schemes. These national financing models, developed in these countries, provide important lessons from successful experiences and common challenges faced by other nations [3, 8, 11]. In this regard, we focus this systematic review on LMICs according to the World Bank’s most recent classification (2021/2022).

Although the last decade has seen a remarkable increase in research on UHC financing issues, few reviews have used a systematic approach, and most have focused on specific geographic regions, such as sub-Saharan Africa and Southeast Asia [12, 13]. Therefore, the value of this study lies in its comprehensive analysis of all interventions related to health financing in LMICs. The goal of this systematic review is to examine the mechanisms for financing universal health coverage in LMICs (revenue collection, pooling of resources, and purchasing), discuss the challenges faced by UHC financing models in these countries, and identify lessons learned from successful experiences for LMICs. To achieve these goals, the results section highlights the primary functions of health financing, while the discussion section addresses key challenges and lessons learned.

## Methods

### Protocol Reporting and Registration

This systematic literature review was carried out following the guidelines of the updated PRISMA 2020 (Preferred Reporting Items for Systematic Reviews and Meta-Analyses) guide [14]. The completed PRISMA checklist is available in the Supplementary Material S1. The review was registered in the PROSPERO international registry of systematic reviews on 08 May 2023, with reference number ID: CRD42023422038.

### Eligibility Criteria

Our eligibility criteria included original scientific studies, both qualitative and quantitative, as well as literature review articles, addressing any or all of the functions of health financing in LMICs with a universal health insurance program to achieve UHC. These functions encompass approaches to fundraising, resource pooling, and the service purchasing function, including provider payment methods and health service packages. We limited the selection to studies published in English and French from 2010, when the WHO released its report on financing universal healthcare, through 2022.

### Information Sources

The literature searches were conducted in November 2022, and seven academic databases were consulted. Notably, PubMed, SCOPUS, WOS, Science Direct, Jstore, Springer, and Cochrane Library. We supplemented this search with an in-depth review of the references of relevant articles identified during the search.

### Search Strategy

Search strategies were developed by the first author (HO) and reviewed by a team of three researchers, including the first author and two co-authors (EB, EA). Searches were carried out using the keywords “Universal health coverage,” “health financing functions,” and “low and middle income countries.” In addition, specific and general MeSH terms were added to capture other relevant articles indexed in the databases. Full search strategies are available in the Supplementary Material S2.

### Selection Process

**FIGURE 1:**
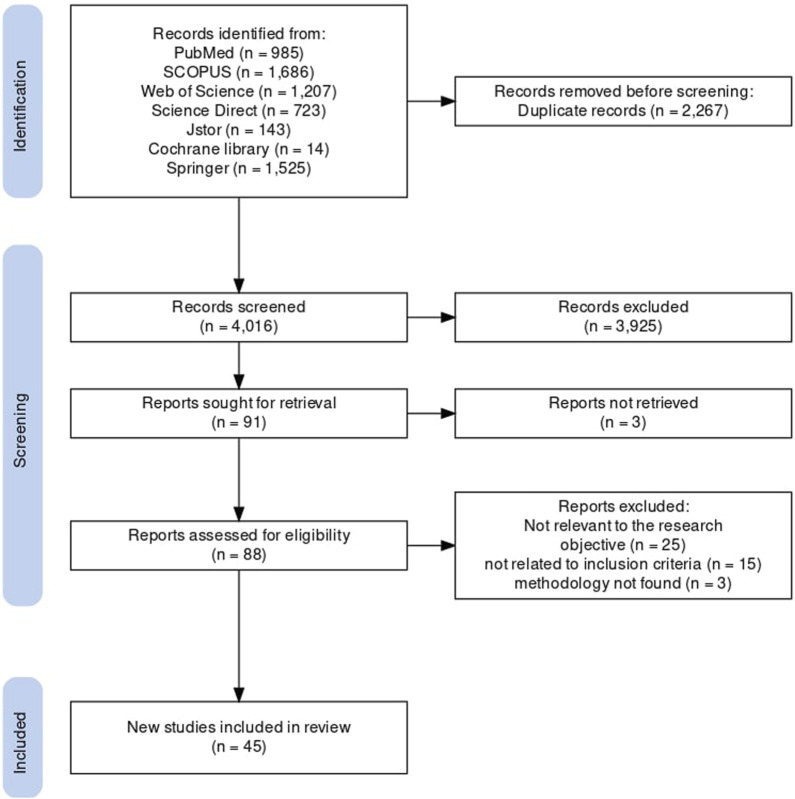
PRISMA flowchart showing the selection and elimination of studies [15] (Systematic review of health financing functions for universal health coverage, Low- and Middle-lncome Countries, 2010–2022).

The article selection process is shown in Figure 1. The electronic search identified 6,283 articles. Records identified in the electronic databases were exported to the bibliographic reference management software “Zotero” for review by the research team.

All duplicate articles (n = 2,267) were removed, and 4,016 articles were retained. Studies were selected in two stages. In the first stage, two reviewers (HO and EB) independently examined the titles and abstracts of the records identified in the searches to confirm their eligibility for the study objective. A total of 91 articles were retained. In a second step, we retrieved the full texts of the articles selected by the two reviewers (HO and EA) to confirm their eligibility based on the inclusion and exclusion criteria. Forty-six articles were then excluded, including 3 for which the full text could not be found. Any disagreements were resolved by consensus. A total of 45 studies met the inclusion criteria for this systematic review and were subsequently included in our qualitative analysis. A summary of the included studies is available in the Supplementary Material S3. No articles were retained by hand-searching the references of articles relevant to our research.

### Data Collection Process

Data were extracted based on the results of studies considered relevant to this review and the essential elements of Kutzin’s (2001) conceptual framework for analyzing national healthcare financing arrangements [11]. Kutzin’s framework identifies three crucial functions of health financing - revenue collection, pooling, and purchasing of healthcare services - which together structure how financial resources are mobilized, managed, and used to progress towards UHC. The two reviewers (HO, EB) independently summarized the data from the included articles, and any disagreements were resolved through discussion between the research team until a consensus was reached. We used a standardized and tested data abstraction form in a spreadsheet using Microsoft Excel, to collect information on authors, year of publication, journal of publication, study setting, population, research objective and question, study design, description of health financing mechanisms, lessons learned, challenges faced, research limitations, conclusions and recommendations.

### Quality Assessment and Risk of Bias

The quality of studies deemed relevant to this study was assessed using the Critical Appraisal Skills Programme (CASP) qualitative checklist, the Joanna Briggs Institute (JBI) assessment tools, and the Mixed Methods Assessment Tool (MMAT). These tools assess and rank studies according to the relevance of their methodological elements (methodological design). They comprise several questions, each with the options “Yes,” “No,” or “Can’t Tell.” We took “Yes” to mean that the study included the answer to the question, and “No” and “Can’t Tell” to mean that the study did not include the answer to the question. Literature review articles, excluding systematic reviews, were excluded from this assessment.

The two reviewers (HO, EB) conducted the assessment independently, and any discrepancies were resolved through consensus. Studies were considered high quality if they met 80% or more of the CASP, JBI, or MMAT criteria, moderate quality if they met a score between 60% and 80%, and low quality if they met a score below 60%. All articles were included in the qualitative analysis, regardless of their final score, given the limited number of articles deemed relevant to our study.

### Data Synthesis Strategy

We synthesized the extracted data using a thematic analysis. The conceptual framework described by Kutzin (2001) was used to derive the initial coding categories. In addition, challenges encountered, potential lessons learned, conclusions, and recommendations were added to the narrative summary. Two reviewers (HO and EA) were involved in this data synthesis process, and any disagreements between them were resolved by discussion between the research team.

### Ethical Statement

This document is a systematic review and therefore does not require consideration of ethical issues.

## Results

### Study Characteristics

**FIGURE 2:**
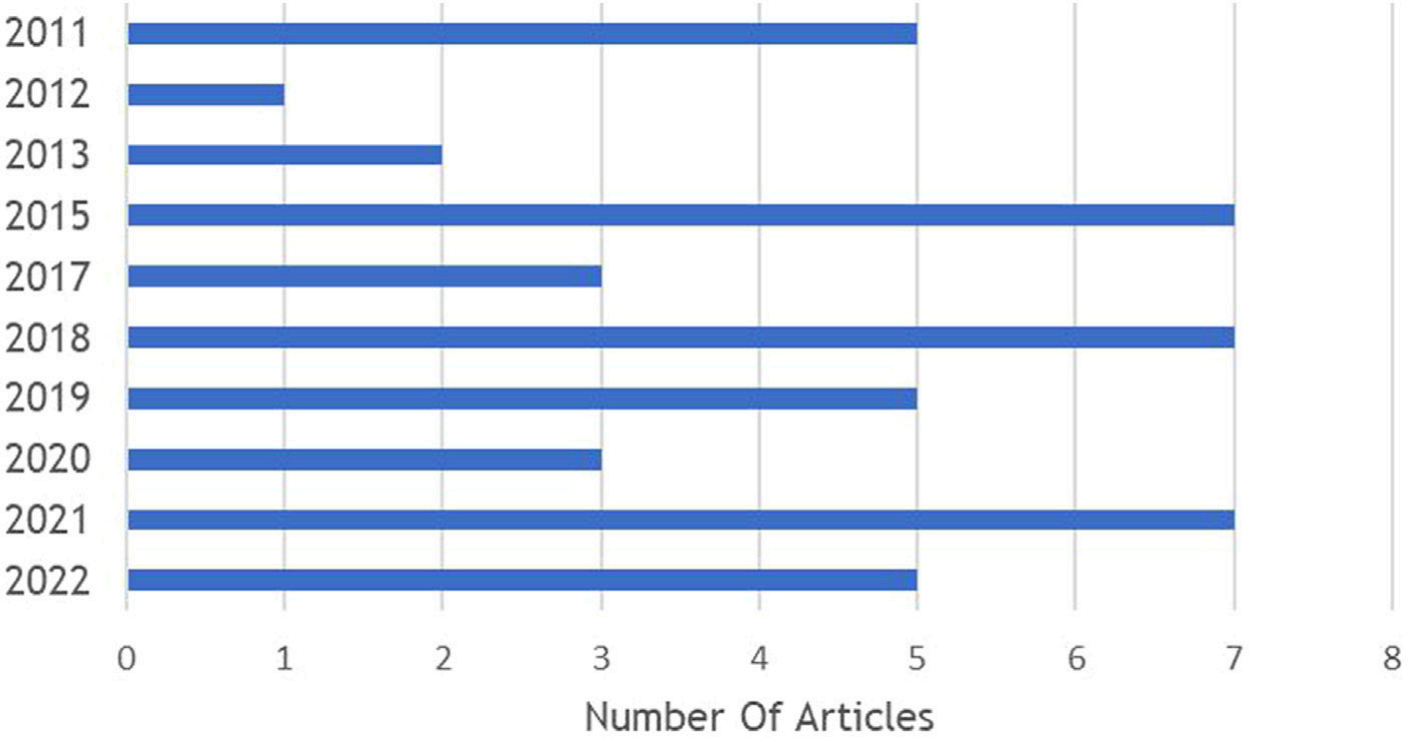
Publication Years (Systematic review of health financing functions for universal health coverage, Low- and Middle-lncome Countries, 2010–2022).

**FIGURE 3:**
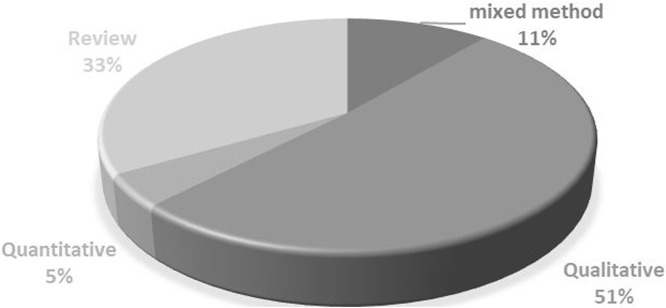
Research Approaches (Systematic review of health financing functions for universal health coverage, Low- and Middle-lncome Countries, 2010–2022).

**FIGURE 4:**
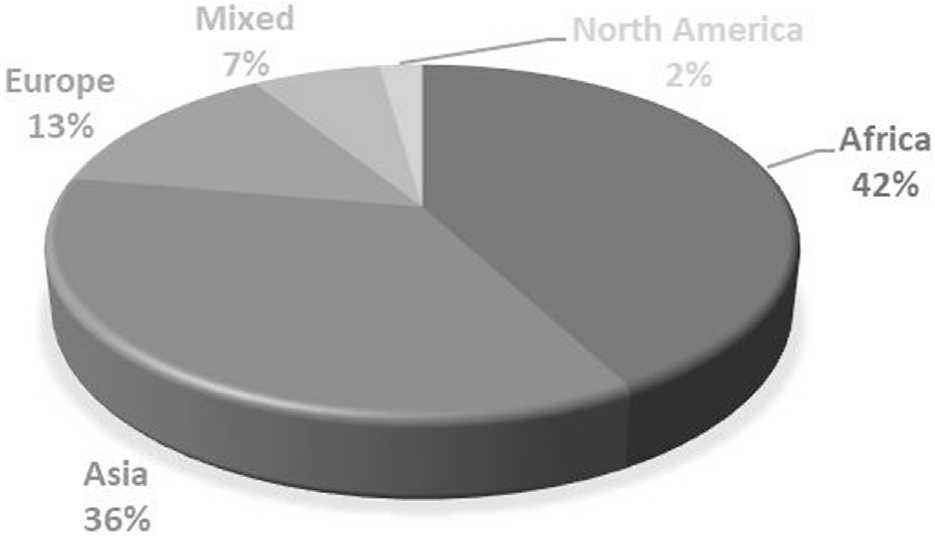
Geographical Distribution (Systematic review of health financing functions for universal health coverage, Low- and Middle-lncome Countries, 2010–2022).

As shown in Figure 2, most articles were published between 2015 and 2022, totaling 37 out of the 45 articles included in this study. In terms of methodology, a trend emerged in favor of qualitative and documentary approaches, with 23 articles and 15 articles, respectively. This trend aligns with the aim of our study, which is to examine financing reforms in depth (Figure 3). Geographically, the majority of studies were carried out in Africa (42%) and Asia (36%), reflecting international efforts to promote universal healthcare in regions with high poverty rates (Figure 4).

### Quality Assessment Results

Details of study quality are provided in the Supplementary Material S4. Although an assessment of study quality was conducted, it was decided not to consider this in interpreting the results because of the exploratory nature of the review. Overall, the studies included in this systematic review showed satisfactory methodological quality. Of the 45 studies included, 18 were rated as high quality, 11 as moderate quality, and 7 as low quality, based on the critical appraisal checklists. Nine review articles were descriptive and were not subjected to formal assessment. Despite differences in quality, all 45 studies were included in the thematic analysis due to their relevance to the review’s objectives and the limited literature available on the topic.

### Fundraising

All health financing systems in the LMICs are considered mixed systems, utilizing financial resources from a variety of public and private sources [12, 16–32]. They utilize financial resources from various sources, including direct payments, taxes, and social health insurance contributions. Private financing of healthcare, characterized primarily by out-of-pocket (OOP) expenditures, plays a significant role in overall healthcare spending in several countries, despite its reputation for being regressive. In India, Nigeria, Nepal, Georgia, Vietnam, and the Philippines, for example, OOP spending is the primary source of healthcare funding [16, 22, 23, 26, 29, 31, 33]. In contrast, countries such as Rwanda, South Africa, Thailand, and Turkey are known for their low share of direct spending in total health expenditure (THE) [18, 19, 24]. As such, external funds, including donors, are an essential source in several LMICs, including Kenya, Nigeria, Rwanda, Tanzania, Cambodia, and Lao PDR [12, 19, 25, 31, 33–35].

In addition to private sources, public prepayment mechanisms, including public taxes and social health insurance, play a major role in healthcare financing in several countries such as Thailand, Turkey, Mexico, Costa Rica and China, which have succeeded in the role of these public sources as the main source of their healthcare financing system [20, 21, 24, 31, 32, 36, 37]. In other countries, such as Ghana [19, 38] and Vietnam [12], their social health insurance schemes rely mainly on earmarked taxes (i.e., indirect taxes).

### Pooling Resources

The structuring of pooled funds for health services in the countries studied takes two main forms. On the one hand, some countries, such as Costa Rica [31], Turkey [39], and Indonesia [18], have merged their various health financing schemes into a single national health fund, financed mainly by public subsidies and member contributions. Similarly, other countries have established a single mutualization system, but one that explicitly includes both contributors and non-contributors in the same risk pool, thereby encouraging cross-subsidies. The latter approach is observed in Ghana, Vietnam, the Philippines, Malaysia, and Northern Macedonia [12, 19, 23, 28, 33].

On the other hand, other countries have multiple pooling systems with different forms of fragmentation. For example, Kenya [40], Nigeria [16], India [18], Iran [2, 24, 41], China [37, 42], Rwanda [19, 43], Tanzania [44], Georgia [29], Cambodia and Lao PDR [12] have fragmented risk-pooling systems based on segmentation of the population according to socio-economic status. In Thailand [21] and Mexico [32], in addition to the various pooling funds for the formal sector, there are other pooling funds for population groups outside the formal sector, with explicit coverage for all population groups. These include Thailand’s Universal Coverage System (UCS) and Mexico’s Social Health Protection System (SPSS), known as Seguro Popular until 2019, before being replaced by the program known as The Health Institute for Wellbeing (INSABI). Another form of risk pooling was observed in the Eastern European countries selected for our study, characterized by regional funds serving the population living in each distinct territory, due to the administrative divisions adopted by each country, including Russia [27] and Bulgaria [30].

### Purchasing of Health Services (Including Provider Payment Methods and Benefit Packages)

Once the funds have been pooled, the final stage in the healthcare financing process is called purchasing. Indeed, the market structure of purchasers of healthcare services in the countries studied falls into two categories, those that use a single purchaser to channel all pooled resources to pay providers on behalf of the entire population, such as the National Health Insurance Scheme (NHIS) in Ghana [18], the Vietnam Social Security (VSS) in Vietnam [23, 45], the Social Security Institution for the Health Sector (BPJS-K) in Indonesia [18], the Social Security Institution (SSI) in Turkey [39], the Social Security Fund (CCSS) in Costa Rica [31], the Health Insurance Fund (HIF) in North Macedonia [28] and the National Health Insurance Fund (NHIF) in Bulgaria [30] through the Regional Health Insurance Fund (RHIF), which constitutes a single purchaser covering the population living in each separate province.

The second model is characterized by the existence of several major purchasers in the same geographical area. There are countries where purchasing is carried out through two mechanisms: integrated purchasing, which generally occurs between the government, via the Minister of Health, and public providers, and contractual purchasing between health insurance schemes and public and/or private providers. Kenya [17, 34, 40, 46–49], Nigeria [35, 50–53], Rwanda [43], Russia [27], and Mexico [32] are examples. Other countries are adopting the demand-driven purchasing model for the entire population through social health insurance schemes. This is the case in China [20, 54], Thailand [21, 31, 55, 56], and Tanzania [44]. In India, private insurance companies act as purchasers of care on behalf of RSBY program members [22]. In Georgia, the Social Services Agency (SSA) purchases healthcare services on behalf of 95% of the population covered by the Universal Health Coverage Program (UHCP), while the wealthiest individuals purchase healthcare services through private insurance companies [29].

Due to the multitude of funding mechanisms, these countries are developing payment systems characterized by a combination of several payment methods, which vary according to the type of healthcare provider, services provided, and coverage scheme. These generally include fee-for-service, global budget, item-based budget, capitation, case-based payment including homogeneous diagnostic groups (DRGs), per diem, and salary. Over the years, various payment methods have been introduced to encourage providers to enhance their performance. In particular, performance-based payment (PBP) has been adopted in Rwanda [33, 43], Turkey [39], and North Macedonia [28].

Regarding the dimensions of the service package, the extent of service coverage in several countries is almost universal. These include the three national schemes in Thailand [31], the Community-Based Health Insurance (CBHI) and Rwanda Social Security Board (RSSB) programs in Rwanda [43], as well as national programs in Vietnam [23], Indonesia [12], and Turkey [36]. However, in Kenya [40], China [20], Tanzania [44], and Russia [27], purchasing organizations offer a varied range of services, differentiated according to the insurer’s status.

## Discussion

This study aims to examine how LMICs have structured their health financing systems to meet UHC objectives. It focuses on the core financing functions—revenue collection, pooling, and purchasing—highlighting the mechanisms employed, the challenges faced, and the strategic choices made. The discussion that follows explores common patterns, context-specific barriers, and promising approaches across various countries.

Despite the considerable variation in regional contexts between LMICs, this analysis was not designed as a regional comparative study due to the uneven distribution of available studies. The discussion is organized around the essential functions of health financing (fundraising, pooling, and purchasing). It also incorporates illustrative examples at the country level to provide contextual information where necessary.

### Fundraising

The studies included in this review often highlight the persistent challenges of resource mobilization, particularly the regressivity of direct payments, which hinders countries’ progress toward UHC [3, 57, 58]. This mechanism is often used as the primary source of funding for many of the countries studied. This observation can be attributed to the low level of public financing in THE, which represents a significant problem in most LMICs. Several studies have demonstrated the significance of public revenues in facilitating rapid progress towards universal healthcare in LMICs [59]. Thanks to government subsidies, China [60] and Thailand [61] have been able to extend medical coverage to populations that did not previously benefit from it. Furthermore, the long-term financial viability of mainly donor-funded health insurance systems in some of the countries studied is questionable [13]. In Cambodia, the Health Equity Fund (HEF) was funded until 2021 in equal parts by the government and donors. However, the donor commitment has come to an end, and responsibility now lies with the government [25]. It is therefore suggested to rely primarily on public funding sources, with a strong commitment from the government.

### Pooling Resources

Concerning risk pooling, the results of our study highlight the high fragmentation of pre-payment systems in LMICs, compounded by limited cross-subsidization between risk pooling funds, leading to the creation of inequalities in access to care, with different levels of financial protection from one risk pool to another, as well as inequities between different territories in the country [12, 27, 31, 62]. Consequently, our results suggest consolidating all fragmented systems into a centralized fund, containing a diversification of health risks to maximize the capacity to redistribute resources. Several LMICs have endeavored to consolidate all fragmented prepayment systems, as shown by the successful experiences of Turkey and Indonesia, which were able to reduce inequalities in access to care by merging all risk pooling funds into a single fund on behalf of the entire population [63, 64]. Other international experiences demonstrate the benefits of consolidating fragmented systems in terms of equity and accessibility to care, control of healthcare expenditure, and savings in administrative costs [65–67]. However, many countries have made efforts to unify their pooling systems, but face several problems, including the large share of the informal sector, as in the case of Tanzania [19], as well as challenges in terms of institutional design and the capacity of administrative and information systems, which still prevent China from unifying its health insurance systems [37].

### Purchasing Health Services

When it comes to healthcare purchasing, a number of challenges hinder active purchasing in the countries studied. Among these challenges, the weakness of institutional and governance arrangements for healthcare purchasing systems is a recurring issue. This weakness manifests itself in the absence of a sound regulatory framework, limited institutional capacity of purchasers, as well as poor budget management reflected in late disbursements and budget overruns. According to Adam Wagstaff (2010), the lack of regulation of national health insurance purchasers has a negative effect on the production of low-cost, high-quality care [68]. In addition, the low capacity of healthcare providers to respond to purchasers’ incentives is generally due to a lack of autonomy in the use of funds.

Other challenges linked to purchasing functions have been identified. Firstly, the absence of a guideline specifying the package of services according to the real needs of the population and national public health priorities, exacerbated by the variability of services offered, given the multiplicity of purchasers, contributes to the creation of inequities. Moreover, contractual agreements are often tacit, regardless of improvements in service quality. This observation aligns with previous work that highlights the lack of selective contracts in LMICs, which limits strategic purchasing [69]. At the same time, all countries use different provider payment systems, given the multiplicity of financing methods, and each payment method has its strengths and weaknesses depending on the context of use. Generally speaking, fee-for-service payment is considered the least strategic, as it can encourage over-consumption of resources and unnecessary practices. Performance-based payment can help motivate staff and improve provider performance, but it can also contribute to inefficiency, as is the case in Turkey [39]. Similarly, performance monitoring mechanisms are often weak, hampering strategic purchasing. In Kenya [47] and Nigeria [50], health data is still paper-based, limiting access to helpful information. In Rwanda [43], information systems are not interoperable, resulting in data duplication. According to Inke Mathauer et al. (2019), many LMICs have difficulty accessing useful health information, compounded by fragmented information systems limiting strategic purchasing [70]. The experience of countries that have successfully transitioned to strategic purchasing has shown significant progress in achieving UHC objectives [12, 13, 24, 28, 31, 39, 55, 56, 71]. This is reflected in the move to a single-payer purchasing model with selective contracts and incentive-based payment methods. These efforts need to be underpinned by strengthened governance, supported by a robust regulatory framework specifying the roles and responsibilities of purchasers and providers separately.

### Study Limitations

This systematic review has certain limitations. First, the published literature tends to focus on specific regions rather than others, with most studies originating from a limited number of countries, including Kenya, Nigeria, China, and Thailand, which could influence our results and conclusions. Second, many studies have concentrated specifically on the third aspect of financing—the purchasing of healthcare services—at the expense of the other two functions, fundraising and resource pooling. Third, most of the studies included in our review are qualitative, likely due to the nature of our research question, which aims to explore in depth the financing mechanisms of universal health coverage in LMICs. Nevertheless, adding more quantitative studies would help provide a clearer picture of the key financing parameters and challenges that directly affect progress toward UHC goals. Finally, the current analysis focused on studies in English and French due to feasibility constraints, which may have excluded relevant data available in other languages used in LMICs.

### Implications for Policy and Research

This study highlights persistent challenges and promising practices in health financing in LMICs. The findings can be a valuable resource for countries seeking to move towards universal health by informing policy decisions on health financing reforms. In addition, this synthesis can guide researchers in identifying unresolved issues related to the core functions of health financing, supporting further investigation and evidence-based strategies to improve the performance and equity of health system financing.

### Conclusion

The results of this systematic review have enabled us to examine in depth the different approaches to financing universal health coverage in LMICs and to identify the main challenges in the field of health financing. The main conclusions drawn are the essential role played by public funds in providing financial protection to the vulnerable population and the extension of medical coverage to all categories, with the government’s share maintained over the long term. Furthermore, the unification of health insurance schemes is viewed as a key factor in promoting equitable access to healthcare services and a fair distribution of resources. In addition, establishing governance with a solid regulatory framework will strengthen the coordination and capabilities of all players involved in purchasing healthcare services. Finally, active purchasing plays a crucial role in improving the performance of the healthcare system, thanks to a selective contract between the purchaser and the provider, encouraging the provider to improve the quality of services in line with pre-established standards, and to control costs through the implementation of payment systems based on the achievement of service delivery targets.
