# Supplementary material for: Systematic Review of Financing Functions for Universal Health Coverage in Low- and Middle-Income Countries: Reforms, Challenges, and Lessons Learned
Source: Public Health Rev. 2025 Sep 23;46:1607745. doi: 10.3389/phrs.2025.1607745 (PMC12501465; doi:10.3389/phrs.2025.1607745)
Supplement: Supplementary file 1 [file Supplementaryfile1.docx]

**Supplemental Material Content**

**A Systematic review of health financing functions for universal health coverage in low- and middle-income countries**

**Supplementary Material 1.** PRISMA 2020 checklist.

**Supplementary Material 2.** Full strategies for each database**.**

**Supplementary Material 3.** Summary of characteristics and findings of the included studies.

**Supplementary Material 4.** Quality Check List.

**Supplementary Material 1.** PRISMA 2020 checklist.

| **Section and Topic** | **Item #** | **Checklist item** | **Location where item is reported** |
| --- | --- | --- | --- |
| **TITLE** | | |  |
| Title | 1 | Identify the report as a systematic review. |  |
| **ABSTRACT** | | |  |
| Abstract | 2 | See the PRISMA 2020 for Abstracts checklist. |  |
| **INTRODUCTION** | | |  |
| Rationale | 3 | Describe the rationale for the review in the context of existing knowledge. |  |
| Objectives | 4 | Provide an explicit statement of the objective(s) or question(s) the review addresses. |  |
| **METHODS** | | |  |
| Eligibility criteria | 5 | Specify the inclusion and exclusion criteria for the review and how studies were grouped for the syntheses. |  |
| Information sources | 6 | Specify all databases, registers, websites, organisations, reference lists and other sources searched or consulted to identify studies. Specify the date when each source was last searched or consulted. |  |
| Search strategy | 7 | Present the full search strategies for all databases, registers and websites, including any filters and limits used. |  |
| Selection process | 8 | Specify the methods used to decide whether a study met the inclusion criteria of the review, including how many reviewers screened each record and each report retrieved, whether they worked independently, and if applicable, details of automation tools used in the process. |  |
| Data collection process | 9 | Specify the methods used to collect data from reports, including how many reviewers collected data from each report, whether they worked independently, any processes for obtaining or confirming data from study investigators, and if applicable, details of automation tools used in the process. |  |
| Data items | 10a | List and define all outcomes for which data were sought. Specify whether all results that were compatible with each outcome domain in each study were sought (e.g. for all measures, time points, analyses), and if not, the methods used to decide which results to collect. |  |
|  | 10b | List and define all other variables for which data were sought (e.g. participant and intervention characteristics, funding sources). Describe any assumptions made about any missing or unclear information. |  |
| Study risk of bias assessment | 11 | Specify the methods used to assess risk of bias in the included studies, including details of the tool(s) used, how many reviewers assessed each study and whether they worked independently, and if applicable, details of automation tools used in the process. |  |
| Effect measures | 12 | Specify for each outcome the effect measure(s) (e.g. risk ratio, mean difference) used in the synthesis or presentation of results. |  |
| Synthesis methods | 13a | Describe the processes used to decide which studies were eligible for each synthesis (e.g. tabulating the study intervention characteristics and comparing against the planned groups for each synthesis (item #5)). |  |
|  | 13b | Describe any methods required to prepare the data for presentation or synthesis, such as handling of missing summary statistics, or data conversions. |  |
|  | 13c | Describe any methods used to tabulate or visually display results of individual studies and syntheses. |  |
|  | 13d | Describe any methods used to synthesize results and provide a rationale for the choice(s). If meta-analysis was performed, describe the model(s), method(s) to identify the presence and extent of statistical heterogeneity, and software package(s) used. |  |
|  | 13e | Describe any methods used to explore possible causes of heterogeneity among study results (e.g. subgroup analysis, meta-regression). |  |
|  | 13f | Describe any sensitivity analyses conducted to assess robustness of the synthesized results. |  |
| Reporting bias assessment | 14 | Describe any methods used to assess risk of bias due to missing results in a synthesis (arising from reporting biases). |  |
| Certainty assessment | 15 | Describe any methods used to assess certainty (or confidence) in the body of evidence for an outcome. |  |
| **RESULTS** | | |  |
| Study selection | 16a | Describe the results of the search and selection process, from the number of records identified in the search to the number of studies included in the review, ideally using a flow diagram. |  |
|  | 16b | Cite studies that might appear to meet the inclusion criteria, but which were excluded, and explain why they were excluded. |  |
| Study characteristics | 17 | Cite each included study and present its characteristics. |  |
| Risk of bias in studies | 18 | Present assessments of risk of bias for each included study. |  |
| Results of individual studies | 19 | For all outcomes, present, for each study: (a) summary statistics for each group (where appropriate) and (b) an effect estimate and its precision (e.g. confidence/credible interval), ideally using structured tables or plots. |  |
| Results of syntheses | 20a | For each synthesis, briefly summarise the characteristics and risk of bias among contributing studies. |  |
|  | 20b | Present results of all statistical syntheses conducted. If meta-analysis was done, present for each the summary estimate and its precision (e.g. confidence/credible interval) and measures of statistical heterogeneity. If comparing groups, describe the direction of the effect. |  |
|  | 20c | Present results of all investigations of possible causes of heterogeneity among study results. |  |
|  | 20d | Present results of all sensitivity analyses conducted to assess the robustness of the synthesized results. |  |
| Reporting biases | 21 | Present assessments of risk of bias due to missing results (arising from reporting biases) for each synthesis assessed. |  |
| Certainty of evidence | 22 | Present assessments of certainty (or confidence) in the body of evidence for each outcome assessed. |  |
| **DISCUSSION** | | |  |
| Discussion | 23a | Provide a general interpretation of the results in the context of other evidence. |  |
|  | 23b | Discuss any limitations of the evidence included in the review. |  |
|  | 23c | Discuss any limitations of the review processes used. |  |
|  | 23d | Discuss implications of the results for practice, policy, and future research. |  |
| **OTHER INFORMATION** | | |  |
| Registration and protocol | 24a | Provide registration information for the review, including register name and registration number, or state that the review was not registered. |  |
|  | 24b | Indicate where the review protocol can be accessed, or state that a protocol was not prepared. |  |
|  | 24c | Describe and explain any amendments to information provided at registration or in the protocol. |  |
| Support | 25 | Describe sources of financial or non-financial support for the review, and the role of the funders or sponsors in the review. |  |
| Competing interests | 26 | Declare any competing interests of review authors. |  |
| Availability of data, code and other materials | 27 | Report which of the following are publicly available and where they can be found: template data collection forms; data extracted from included studies; data used for all analyses; analytic code; any other materials used in the review. |  |

**Supplementary Material 2.** Full strategies for each database

“The literature searches were carried out in November 2022 in seven academic databases. Each database has specific technical features and limitations that influenced the structure of the search strategy. Some databases enable more advanced Boolean operations and allow the inclusion of a wide range of synonyms and MeSH terms. On the other hand, other databases impose restrictions on the number of concepts or characters allowed per search query. These differences explain the variation in length and structure of search strategies between databases..”

**Search in PubMed :**

((("Healthcare financing"[MeSH Terms]) OR (("health financ*"[Title/Abstract]) OR ("healthcare financ*"[Title/Abstract]) OR ("health financing arrangement*"[Title/Abstract]) OR ("health financing mechanism*"[Title/Abstract]) OR ("Health financing strateg*"[Title/Abstract]) OR ("Health financing approach*"[Title/Abstract]) OR ("Health financing function*"[Title/Abstract]) OR ("Health financing system*"[Title/Abstract]) OR ("Revenue collection"[Title/Abstract]) OR ("Mandatory prepayment"[Title/Abstract]) OR ("Compulsory prepayment"[Title/Abstract]) OR ("General revenues"[Title/Abstract]) OR ("Direct taxes"[Title/Abstract]) OR ("Indirect Taxes"[Title/Abstract]) OR ("Taxes"[Title/Abstract]) OR ("compulsory insurance"[Title/Abstract]) OR ("privately funded"[Title/Abstract] ) OR ("Out-of-pocket"[Title/Abstract]) OR ("foreign fund*"[Title/Abstract]) OR ("Fund pooling"[Title/Abstract]) OR ("Purchasing"[Title/Abstract]) OR ("service benefits"[Title/Abstract]) OR ("payment mechanism"[Title/Abstract]) OR ("fee-for-service"[Title/Abstract]) OR ("capitation"[Title/Abstract]) OR ("global budget"[Title/Abstract]) OR ("salary"[Title/Abstract]) OR ("fee schedules"[Title/Abstract]) OR ("DRGs"[Title/Abstract]))) AND ((("Universal Health Insurance"[Mesh]) OR ("Universal Health Care"[Mesh])) OR (("universal health coverage"[Title/Abstract]) OR (universal AND health AND coverage[Title/Abstract]) OR (Universal AND health*[Title/Abstract]) OR ("Universal health*"[Title/Abstract]) OR ("couverture sanitaire universelle"[Title/Abstract]) OR ("health coverage"[Title/Abstract]) OR ("universal coverage"[Title/Abstract]) OR ("universal health"[Title/Abstract]) OR ("uhc"[Title/Abstract]) OR ("universal health care"[Title/Abstract]) OR ("universal care"[Title/Abstract]) OR ("universal health insurance"[Title/Abstract]) OR ("universal access"[Title/Abstract]) OR ("social health insurance"[Title/Abstract]) OR ("socialized healthcare"[Title/Abstract]) OR ("Social health protection "[Title/Abstract]) OR ("Social welfare"[Title/Abstract]) OR ("health equity"[Title/Abstract]) OR ("social protection in health"[Title/Abstract]) OR ("social security"[Title/Abstract]) OR ("social security"[Title/Abstract]))) AND ((("Latin America"[Mesh]) OR ("Pacific Islands"[Mesh]) OR ("Developing Countries"[Mesh]) OR ("Africa"[Mesh]) OR ("Asia, Northern"[Mesh]) OR ("Asia, Western"[Mesh]) OR ("Asia, Southeastern"[Mesh]) OR ("Asia, Central"[Mesh]) OR ("Europe, Eastern"[Mesh])) OR ("low and middle income countries"[Title/Abstract] OR "LMIC"[Title/Abstract] OR "developing countr*"[Title/Abstract] OR "Afghanistan"[Title/Abstract] OR "Guinea-Bissau"[Title/Abstract] OR "Somalia"[Title/Abstract] OR "Burkina Faso"[Title/Abstract] OR "the Democratic People's Republic of Korea"[Title/Abstract] OR "North Korea"[Title/Abstract] OR "South Sudan"[Title/Abstract] OR "Burundi"[Title/Abstract] OR "Liberia"[Title/Abstract] OR "Sudan"[Title/Abstract] OR "Central African Republic"[Title/Abstract] OR "Madagascar"[Title/Abstract] OR "Syrian Arab Republic"[Title/Abstract] OR "Chad"[Title/Abstract] OR "Malawi"[Title/Abstract] OR "Togo"[Title/Abstract] OR "Congo, Dem. Rep"[Title/Abstract] OR " Democratic Republic of the Congo"[Title/Abstract] OR "Mali"[Title/Abstract] OR "Uganda"[Title/Abstract] OR "Eritrea"[Title/Abstract] OR "Mozambique"[Title/Abstract] OR "Yemen, Rep."[Title/Abstract] OR "Yemen"[Title/Abstract] OR "Ethiopia"[Title/Abstract] OR "Niger"[Title/Abstract] OR "Zambia"[Title/Abstract] OR "Gambia, The"[Title/Abstract] OR "Rwanda"[Title/Abstract] OR "Guinea"[Title/Abstract] OR "Sierra Leone"[Title/Abstract] OR "Angola"[Title/Abstract] OR "India"[Title/Abstract] OR "Philippines"[Title/Abstract] OR "Algeria"[Title/Abstract] OR "Indonesia"[Title/Abstract] OR "Samoa"[Title/Abstract] OR "Bangladesh"[Title/Abstract] OR "Iran, Islamic Rep"[Title/Abstract] OR "Iran"[Title/Abstract] OR " the Islamic Republic of Iran"[Title/Abstract] OR "Sao Tome and Principe"[Title/Abstract] OR "Benin"[Title/Abstract] OR "Kenya"[Title/Abstract] OR "Senegal"[Title/Abstract] OR "Bhutan"[Title/Abstract] OR "Kiribati"[Title/Abstract] OR "Solomon Islands"[Title/Abstract] OR "Bolivia"[Title/Abstract] OR "Kyrgyz Republic"[Title/Abstract] OR "Sri Lanka"[Title/Abstract] OR "Cabo Verde"[Title/Abstract] OR "Lao PDR"[Title/Abstract] OR "Tanzania"[Title/Abstract] OR "Cambodia"[Title/Abstract] OR "Lebanon"[Title/Abstract] OR "Tajikistan"[Title/Abstract] OR "Cameroon"[Title/Abstract] OR "Lesotho"[Title/Abstract] OR "Timor-Leste"[Title/Abstract] OR "Comoros"[Title/Abstract] OR "Mauritania"[Title/Abstract] OR "Tunisia"[Title/Abstract] OR "Congo, Rep."[Title/Abstract] OR "Republic of the Congo"[Title/Abstract] OR "Congo-Brazzaville"[Title/Abstract] OR "Micronesia, Fed. Sts."[Title/Abstract] OR "The Federated States of Micronesia"[Title/Abstract] OR "Micronesia"[Title/Abstract] OR "Ukraine"[Title/Abstract] OR "Cote d'Ivoire"[Title/Abstract] OR "Mongolia"[Title/Abstract] OR "Uzbekistan"[Title/Abstract] OR "Djibouti"[Title/Abstract] OR "Morocco"[Title/Abstract] OR "Vanuatu"[Title/Abstract] OR "Egypt, Arab Rep."[Title/Abstract] OR "Arab Republic of Egypt"[Title/Abstract] OR "Egypt"[Title/Abstract] OR "Myanmar"[Title/Abstract] OR "Vietnam"[Title/Abstract] OR "El Salvador"[Title/Abstract] OR "Nepal"[Title/Abstract] OR "West Bank and Gaza"[Title/Abstract] OR "Eswatini"[Title/Abstract] OR "Nicaragua"[Title/Abstract] OR "Zimbabwe"[Title/Abstract] OR "Ghana"[Title/Abstract] OR "Nigeria"[Title/Abstract] OR "Haiti"[Title/Abstract] OR "Pakistan"[Title/Abstract] OR "Honduras"[Title/Abstract] OR "Papua New Guinea"[Title/Abstract] OR "Albania"[Title/Abstract] OR "Fiji"[Title/Abstract] OR "Namibia"[Title/Abstract] OR "American Samoa"[Title/Abstract] OR "Gabon"[Title/Abstract] OR "North Macedonia"[Title/Abstract] OR "Argentina"[Title/Abstract] OR "Georgia"[Title/Abstract] OR "Palau"[Title/Abstract] OR "Armenia"[Title/Abstract] OR "Grenada"[Title/Abstract] OR "Paraguay"[Title/Abstract] OR "Azerbaijan"[Title/Abstract] OR "Guatemala"[Title/Abstract] OR "Peru"[Title/Abstract] OR "Belarus"[Title/Abstract] OR "Guyana"[Title/Abstract] OR "Russian Federation"[Title/Abstract] OR "Belize"[Title/Abstract] OR "Iraq"[Title/Abstract] OR "Serbia"[Title/Abstract] OR "Bosnia and Herzegovina"[Title/Abstract] OR "Jamaica"[Title/Abstract] OR "South Africa"[Title/Abstract] OR "Botswana"[Title/Abstract] OR "Jordan"[Title/Abstract] OR "St. Lucia"[Title/Abstract] OR "Brazil"[Title/Abstract] OR "Kazakhstan"[Title/Abstract] OR "St. Vincent and the Grenadines"[Title/Abstract] OR "Bulgaria"[Title/Abstract] OR "Kosovo"[Title/Abstract] OR "Suriname"[Title/Abstract] OR "China"[Title/Abstract] OR "Libya"[Title/Abstract] OR "Thailand"[Title/Abstract] OR "Colombia"[Title/Abstract] OR "Malaysia"[Title/Abstract] OR "Tonga"[Title/Abstract] OR "Costa Rica"[Title/Abstract] OR "Maldives"[Title/Abstract] OR "Turkiye"[Title/Abstract] OR "Cuba"[Title/Abstract] OR "Marshall Islands"[Title/Abstract] OR "Turkmenistan"[Title/Abstract] OR "Dominica"[Title/Abstract] OR "Mauritius"[Title/Abstract] OR "Tuvalu"[Title/Abstract] OR "Dominican Republic"[Title/Abstract] OR "Mexico"[Title/Abstract] OR "Equatorial Guinea"[Title/Abstract] OR "Moldova"[Title/Abstract] OR "Ecuador"[Title/Abstract] OR "Montenegro"[Title/Abstract]))) AND ((humans[Filter]) AND (english[Filter] OR french[Filter]) AND (2010:2022[pdat])).

**Search in SCOPUS :**

TITLE-ABS-KEY ( ( "health financ*" OR "healthcare financ*" OR "health financing arrangement*" OR "health financing mechanism*" OR "Health financing strateg*" OR "Health financing approach*" OR "Health financing function*" OR "Health financing system*" OR "Revenue collection" OR "Mandatory prepayment" OR "Compulsory prepayment" OR "General revenues" OR "Direct taxes" OR "Indirect Taxes" OR "Taxes" OR "compulsory insurance" OR "privately funded" OR "Out-of-pocket" OR "foreign fund*" OR "Fund pooling" OR "Purchasing" OR "service benefits" OR "payment mechanism" OR "fee-for-service" OR "capitation" OR "global budget" OR "salary" OR "fee schedules" OR "DRGs" ) AND ( "universal health coverage" OR ( universal AND health AND coverage ) OR ( universal AND health* ) OR "Universal health*" OR "couverture sanitaire universelle" OR "health coverage" OR "universal coverage" OR "universal health" OR ( uhc ) OR "universal health care" OR "universal care" OR "universal health insurance" OR "universal access" OR "social health insurance" OR "socialized healthcare " OR "Social health protection " OR "Social welfare" OR "health equity" OR "social protection in health" OR "social security" ) AND ( "low and middle income countries" OR "LMIC" OR "developing countr*" OR "Afghanistan" OR "Guinea-Bissau" OR "Somalia" OR "Burkina Faso" OR "the Democratic People's Republic of Korea" OR "North Korea" OR "South Sudan" OR "Burundi" OR "Liberia" OR "Sudan" OR "Central African Republic" OR "Madagascar" OR "Syrian Arab Republic" OR "Chad" OR "Malawi" OR "Togo" OR "Congo, Dem. Rep" OR " Democratic Republic of the Congo" OR "Mali" OR "Uganda" OR "Eritrea" OR "Mozambique" OR "Yemen, Rep." OR "Yemen" OR "Ethiopia" OR "Niger" OR "Zambia" OR "Gambia, The" OR "Rwanda" OR "Guinea" OR "Sierra Leone" OR "Angola" OR "India" OR "Philippines" OR "Algeria" OR "Indonesia" OR "Samoa" OR "Bangladesh" OR "Iran, Islamic Rep" OR "Iran" OR " the Islamic Republic of Iran" OR "São Tomé and Principe" OR "Benin" OR "Kenya" OR "Senegal" OR "Bhutan" OR "Kiribati" OR "Solomon Islands" OR "Bolivia" OR "Kyrgyz Republic" OR "Sri Lanka" OR "Cabo Verde" OR "Lao PDR" OR "Tanzania" OR "Cambodia" OR "Lebanon" OR "Tajikistan" OR "Cameroon" OR "Lesotho" OR "Timor-Leste" OR "Comoros" OR "Mauritania" OR "Tunisia" OR "Congo, Rep." OR "Republic of the Congo" OR "Congo-Brazzaville" OR "Micronesia, Fed. Sts." OR "The Federated States of Micronesia" OR "Micronesia" OR "Ukraine" OR "Côte d'Ivoire" OR "Mongolia" OR "Uzbekistan" OR "Djibouti" OR "Morocco" OR "Vanuatu" OR "Egypt, Arab Rep." OR "Arab Republic of Egypt" OR "Egypt" OR "Myanmar" OR "Vietnam" OR "El Salvador" OR "Nepal" OR "West Bank and Gaza" OR "Eswatini" OR "Nicaragua" OR "Zimbabwe" OR "Ghana" OR "Nigeria" OR "Haiti" OR "Pakistan" OR "Honduras" OR "Papua New Guinea" OR "Albania" OR "Fiji" OR "Namibia" OR "American Samoa" OR "Gabon" OR "North Macedonia" OR "Argentina" OR "Georgia" OR "Palau" OR "Armenia" OR "Grenada" OR "Paraguay" OR "Azerbaijan" OR "Guatemala" OR "Peru" OR "Belarus" OR "Guyana" OR "Russian Federation" OR "Belize" OR "Iraq" OR "Serbia" OR "Bosnia and Herzegovina" OR "Jamaica" OR "South Africa" OR "Botswana" OR "Jordan" OR "St. Lucia" OR "Brazil" OR "Kazakhstan" OR "St. Vincent and the Grenadines" OR "Bulgaria" OR "Kosovo" OR "Suriname" OR "China" OR "Libya" OR "Thailand" OR "Colombia" OR "Malaysia" OR "Tonga" OR "Costa Rica" OR "Maldives" OR "Türkiye" OR "Cuba" OR "Marshall Islands" OR "Turkmenistan" OR "Dominica" OR "Mauritius" OR "Tuvalu" OR "Dominican Republic" OR "Mexico" OR "Equatorial Guinea" OR "Moldova" OR "Ecuador" OR "Montenegro" )) AND DOCTYPE ( ar OR re ) AND ( PUBYEAR > 2009 AND PUBYEAR < 2023 ) . AND ( LIMIT-TO ( SRCTYPE , "j" ) ) AND ( LIMIT-TO ( PUBSTAGE , "final" ) ) AND ( LIMIT-TO ( LANGUAGE , "English" ) OR LIMIT-TO ( LANGUAGE , "French" ) ).

**Search in WOS :**

((TS=(( "health financ*" OR "healthcare financ*" OR "health financing arrangement*" OR "health financing mechanism*" OR "Health financing strateg*" OR "Health financing approach*" OR "Health financing function*" OR "Health financing system*" OR "Revenue collection" OR "Mandatory prepayment" OR "Compulsory prepayment" OR "General revenues" OR "Direct taxes" OR "Indirect Taxes" OR "Taxes" OR "compulsory insurance" OR "privately funded" OR "Out-of-pocket" OR "foreign fund*" OR "Fund pooling" OR "Purchasing" OR "service benefits" OR "payment mechanism" OR "fee-for-service" OR "capitation" OR "global budget" OR "salary" OR "fee schedules" OR "DRGs" ) )) AND TS=(( "universal health coverage" OR ( universal AND health AND coverage ) OR ( universal AND health* ) OR "Universal health*" OR "couverture sanitaire universelle" OR "health coverage" OR "universal coverage" OR "universal health" OR ( uhc ) OR "universal health care" OR "universal care" OR "universal health insurance" OR "universal access" OR "social health insurance" OR "socialized healthcare " OR "Social health protection " OR "Social welfare" OR "health equity" OR "social protection in health" OR "social security" ))) AND TS=(( "low and middle income countries" OR "LMIC" OR "developing countr*" OR "Afghanistan" OR "Guinea-Bissau" OR "Somalia" OR "Burkina Faso" OR "the Democratic People's Republic of Korea" OR "North Korea" OR "South Sudan" OR "Burundi" OR "Liberia" OR "Sudan" OR "Central African Republic" OR "Madagascar" OR "Syrian Arab Republic" OR "Chad" OR "Malawi" OR "Togo" OR "Congo, Dem. Rep" OR " Democratic Republic of the Congo" OR "Mali" OR "Uganda" OR "Eritrea" OR "Mozambique" OR "Yemen, Rep." OR "Yemen" OR "Ethiopia" OR "Niger" OR "Zambia" OR "Gambia, The" OR "Rwanda" OR "Guinea" OR "Sierra Leone" OR "Angola" OR "India" OR "Philippines" OR "Algeria" OR "Indonesia" OR "Samoa" OR "Bangladesh" OR "Iran, Islamic Rep" OR "Iran" OR " the Islamic Republic of Iran" OR "São Tomé and Principe" OR "Benin" OR "Kenya" OR "Senegal" OR "Bhutan" OR "Kiribati" OR "Solomon Islands" OR "Bolivia" OR "Kyrgyz Republic" OR "Sri Lanka" OR "Cabo Verde" OR "Lao PDR" OR "Tanzania" OR "Cambodia" OR "Lebanon" OR "Tajikistan" OR "Cameroon" OR "Lesotho" OR "Timor-Leste" OR "Comoros" OR "Mauritania" OR "Tunisia" OR "Congo, Rep." OR "Republic of the Congo" OR "Congo-Brazzaville" OR "Micronesia, Fed. Sts." OR "The Federated States of Micronesia" OR "Micronesia" OR "Ukraine" OR "Côte d'Ivoire" OR "Mongolia" OR "Uzbekistan" OR "Djibouti" OR "Morocco" OR "Vanuatu" OR "Egypt, Arab Rep." OR "Arab Republic of Egypt" OR "Egypt" OR "Myanmar" OR "Vietnam" OR "El Salvador" OR "Nepal" OR "West Bank and Gaza" OR "Eswatini" OR "Nicaragua" OR "Zimbabwe" OR "Ghana" OR "Nigeria" OR "Haiti" OR "Pakistan" OR "Honduras" OR "Papua New Guinea" OR "Albania" OR "Fiji" OR "Namibia" OR "American Samoa" OR "Gabon" OR "North Macedonia" OR "Argentina" OR "Georgia" OR "Palau" OR "Armenia" OR "Grenada" OR "Paraguay" OR "Azerbaijan" OR "Guatemala" OR "Peru" OR "Belarus" OR "Guyana" OR "Russian Federation" OR "Belize" OR "Iraq" OR "Serbia" OR "Bosnia and Herzegovina" OR "Jamaica" OR "South Africa" OR "Botswana" OR "Jordan" OR "St. Lucia" OR "Brazil" OR "Kazakhstan" OR "St. Vincent and the Grenadines" OR "Bulgaria" OR "Kosovo" OR "Suriname" OR "China" OR "Libya" OR "Thailand" OR "Colombia" OR "Malaysia" OR "Tonga" OR "Costa Rica" OR "Maldives" OR "Türkiye" OR "Cuba" OR "Marshall Islands" OR "Turkmenistan" OR "Dominica" OR "Mauritius" OR "Tuvalu" OR "Dominican Republic" OR "Mexico" OR "Equatorial Guinea" OR "Moldova" OR "Ecuador" OR "Montenegro" )). Refined By : Document types : Article or Review article, Languages:French or English, Publication Years : From 2010 To 2022.

**Search in ScienceDirect :**

("health financing mechanisms" OR "revenue collection" OR "fund pooling" OR "purchasing") AND ("universal health coverage" OR "universal coverage") AND ("Developing Countries" OR "low income countries" OR "middle income countries") Year: 2010-2022, Article types : review articles or research articles

**Search in Jstor :**

("health financing mechanisms" OR "revenue collection" OR "fund pooling" OR "purchasing")

AND

("universal health coverage" OR "universal coverage")

AND

("devoloping countries" OR "low income countries" OR "middle income countries")

Limits : PUBLICATION DATE :from 2010 to 2022, ITEM TYPE :articls or reviews

**Search in Cochrane library:**

#1 MeSH descriptor: [Healthcare Financing] explode all trees 8

#2 (( "health financ*" OR "healthcare financ*" OR "health financing arrangement*" OR "health financing mechanism*" OR "Health financing strateg*" OR "Health financing approach*" OR "Health financing function*" OR "Health financing system*" OR "Revenue collection" OR "Mandatory prepayment" OR "Compulsory prepayment" OR "General revenues" OR "Direct taxes" OR "Indirect Taxes" OR "Taxes" OR "compulsory insurance" OR "privately funded" OR "Out-of-pocket" OR "foreign fund*" OR "Fund pooling" OR "Purchasing" OR "service benefits" OR "payment mechanism" OR "fee-for-service" OR "capitation" OR "global budget" OR "salary" OR "fee schedules" OR "DRGs" )):ti,ab,kw (Word variations have been searched) with Cochrane Library publication date Between Jan 2010 and Nov 2022, in Cochrane Reviews, Trials 5396

#3 MeSH descriptor: [Universal Health Insurance] explode all trees 5

#4 (( "universal health coverage" OR ( universal AND health AND coverage ) OR ( universal AND health* ) OR "Universal health*" OR "couverture sanitaire universelle" OR "health coverage" OR "universal coverage" OR "universal health" OR ( uhc ) OR "universal health care" OR "universal care" OR "universal health insurance" OR "universal access" OR "social health insurance" OR "socialized healthcare " OR "Social health protection " OR "Social welfare" OR "health equity" OR "social protection in health" OR "social security" )):ti,ab,kw (Word variations have been searched) with Cochrane Library publication date Between Jan 2010 and Nov 2022, in Cochrane Reviews, Trials 21700

#5 MeSH descriptor: [Developing Countries] explode all trees 917

#6 (( "low and middle income countries" OR "LMIC" OR "developing countr*" OR "low income countr*" OR "middle income countr*")):ti,ab,kw (Word variations have been searched) with Cochrane Library publication date Between Jan 2010 and Nov 2022, in Cochrane Reviews, Trials 1684

#7 #1 OR #2 5396

#8 #3 OR #4 21702

#9 #5 OR #6 2482

#10 #7 AND #8 AND #9 14

**Search in Springer :**

'("health financ*" OR "healthcare financ*" OR "health financing arrangement*" OR "health financing mechanism*" OR "Health financing approach*" OR "Health financing function*" OR "Revenue collection" OR "Fund pooling" OR "Purchasing") AND ( "universal health coverage" OR "universal coverage" OR "universal health insurance") AND ("low and middle income countries" OR "low income countries" OR "middle income countries" OR "developing countries")'

within Article, 2010 - 2022

**Supplemental Material 3.** Summary of characteristics and findings of the included studies

| **REf.** | **Year/ method/ study setting** | **Main finding:**  **Health financing mechanisms, challenges and key lessons learned** |
| --- | --- | --- |
| R. Mbau et al [47] | 2018/ Qualitative case study/ Kenya | ***Health financing mechanisms:***  -In Kenya, healthcare services are purchased according to two models: the contractual model, where the National Hospital Insurance Fund and private insurers enter into contracts with public and private healthcare facilities, and the integrated model, where the Ministry of Health and county health departments purchase services directly from the public hospitals they own, according to their level of care.  ***Challenges:***  -lack of adequate funding, weak institutional capacity and lack of technical expertise. |
| K. Munge et al [34] | 2019/ Qualitative case study/ kenya | ***Health financing mechanisms:***  -Healthcare is financed mainly by private sources (Out of pocket), followed by government sources and donors.  -The provider payment mechanism used by both Micro health insurance(MHIs) was fee-for-service.  ***Challenges:***  -The challenges include poor coordination among government bodies, unclear communication between insured individuals and purchasers, weak financial protection, and the absence of guidelines for contribution rates, benefits, and provider selection. |
| K. Munge et al [48] | 2018/ Qualitative case study/ kenya | ***Health financing mechanisms:***  - the main sources of health funding are public spending (31%), private spending (40%) and donor spending (25%).  - healthcare is purchased through three mechanisms: government entities purchase services from the public health facilities they own, the National Hospital Insurance Fund contracts with public and private providers to serve registered members, and private and community health insurance schemes contract with providers to supply services to their members.  ***Challenges:***  -lack of transparency in the healthcare procurement process, the weak capacity of healthcare providers, and the absence of a solid regulatory and policy framework. |
| H. Ibrahimipour et al [2] | 2011/Qualitative descriptive cross-sectional study/ Iran | ***Challenges:***  Several significant challenges confront the healthcare system, including a lack of information, regressive financing, fragmented risk pools, the absence of standardized benefit packages, and the fee-for-service payment system |
| C.-Y. Myint et al [12] | 2019/Systematic review /Brunei, Cambodia, Indonesia, Lao PDR, Malaysia, Myanmar, Philippines, Singapore, Thailand and Viet Nam, China | ***Health financing mechanisms:***  the main sources of revenue for healthcare are direct/indirect taxes and out-of-pocket payments(OOP). Some countries have unified health revenue pooling systems, which feed into a single national health insurance fund. Others employ more complex pooling systems involving several insurance schemes and diverse funding sources. Each country defines its basic service offering, including essential health services. Payment methods for healthcare providers include capitation, fee-for-service, diagnosis-related groups (DRGs), tariffs, salaries and global budgets.  ***Challenges:***  Low overall and government healthcare spending, combined with increasing healthcare demand and costs, pose significant challenges. Fragmented financing systems contribute to disparities in healthcare access. Fee-for-service payments may strain the financial sustainability of community-based insurance (CBI) schemes, while capitation payments could compromise quality care delivery.  ***key lessons learned:***  Strong political will to invest in healthcare and public investment are essential to achieve universal health coverage (UHC). Consolidating fragmented health insurance schemes can improve efficiency and equity. Single-payer models offer equitable access, while capitation payments can improve the efficiency of health insurance systems in ASEAN countries. The integration of accreditation programs into health insurance systems is essential to improve the quality of services. |
| V. Tangcharoensathien et al [55] | 2015/ Qualitative study/ Thailand | ***Health financing mechanisms:***  Members of UCS program receive a tailored basket of services to meet their needs, with a focus on accrediting healthcare providers. Two distinct payment methods are employed: capitation and a national global budget, within which the relative weights of DRGs are utilized for national discharges.  ***Challenges:***  fee-for-service payment for outpatient services leads to over-utilization of resources and high costs  ***key lessons learned:***  Capitation payment under Universal Health Coverage (UHC) helps control costs. The global budget and Diagnostic Related Groups (DRG) effectively contain expenses. The National Health Security Office (NHSO) wields monopsonistic purchasing power as a single buyer. The global budget helps mitigate financial implications of DRG shifting. |
| M. K. Domapielle [38] | 2021/ Qualitive study/Ghana | ***Health financing mechanisms:***  The main sources of revenue for the National Health Insurance Scheme (NHIS) include tax revenues, and premium payments by members of the informal sector. These resources were subsequently pooled into a single fund: the National Health Insurance Fund (NHIF).  ***Challenges:***  The formalization of direct payments and co-payments for benefits covered by the NHIS stems from delays in claims reimbursement and inadequate reimbursement rates. |
| A. Kuwawenaruwa [44] | 2022/ multi-case qualitative study /Tanzania | ***Health financing mechanisms:***  -NHIF (National Health Insurance Fund): Employee contributions/Voluntary contributions. SHIB (Social Health Insurance Benefit): Employee and employer contributions: SHIB is managed by the National Social Security Fund (NSSF). iCHF (Improved Community Health Fund): Voluntary contributions.  - funds are pooled at national level for NHIF and SHIB, and at regional level for iCHF.  - The NHIF provides a wide range of services, with automatically accredited public providers and agreements with some private providers. Provider payment is on a fee-for-service basis. The SHIB and iCHF respectively offer inpatient and outpatient care on a capitation basis, and primary healthcare services on a capitation basis. All three schemes have control mechanisms to evaluate provider performance.  ***Challenges:***  The NHIF offers a wide choice of providers and benefits, compared with plans limited in providers. However, the fee-for-service approach used by the NHIF can lead to inefficiencies. Similarly, the capitation system used by SHIB can impact service quality and create disparities in access. |
| N. K. Phuong [45] | 2015/ Qualitative assessment study/ Vietnam | ***Health financing mechanisms:***  the provider payment system in Vietnam includes several modalities, such as global budgets, line item budgets, fee-for-service and capitation.  ***Challenges:***  Insufficient funding of the budgetary system compromises its contribution to quality of care. Fee-for-service payment can encourage excessive provision of costly services. Capitation is less favored to ensure equity and equitable distribution of resources. |
| A. Dimova et al [30] | 2018/Review/Bulgaria | ***Health financing mechanisms:***  Health care is financed by compulsory Social Security contributions (SHI), taxes, out-of-pocket payments, Voluntary Health Insurance (VHI) premiums and other sources. The National Health Insurance Fund (NHIF) allocates funds to the 28 Regional Health Insurance Funds (RHIFs) according to population and estimated health needs. As the main purchaser of healthcare services, the NHIF uses a contractual model with providers, remunerating them through various methods such as fee-for-service, per capita and global budgets.  ***Challenges:***  high level of OOP |
| Q. Meng et al [37] | 2015/ Review/ China | ***Health financing mechanisms:***  Payroll taxes are the main source of funding for the Urban Employee Basic Medical Insurance (UEBMI), and government subsidies are the main sources of funding for the New Cooperative Medical Scheme (NCMS) and the Urban Resident Basic Medical Insurance (URBMI). Regarding pooling, NCMS funds are pooled at the county level, and URBMI and UEBMI funds are pooled at the municipal (prefecture) level.  ***Challenges:***  weak institutional, administrative and information system capacities.  ***key lessons learned:***  The consolidation of healthcare insurance systems in various countries has shown positive improvements in population coverage, healthcare access, reduction of financial burdens on low-income families, and administrative cost reduction. These results are attributed to factors like political commitment, legislation, public financial support, and administrative organization. |
| C. Ezenduka et al [51] | 2022/Qualitative, descriptive case study/Nigeria | ***Health financing mechanisms:***  The State Ministry of Health (SMOH) is Imo State's main healthcare provider and largest purchaser of healthcare services. The newly created Imo State Health Insurance Agency (IMSHIA) is the second largest purchaser, remunerating providers through capitation and fee-for-service models. IMSHIA offers a comprehensive healthcare package tailored to the state's needs, covering promotion, prevention, curative care and rehabilitation.  ***Challenges:***  An insufficient framework for Health Purchasing System (HPS), lack of accountability among purchasers, absence of separation between purchasers and service providers, and inadequate performance monitoring and payment mechanisms hinder the promotion of quality and efficiency in service provision. |
| R. Mbau et al [46] | 2020/Qualitive case study/Kenya | ***Health financing mechanisms:***  The NHIF uses a variety of methods to pay providers, such as capitalization for ambulatory services, case-based and fee-for-service payment for specialized services, and per diem payment.  ***Challenges:***  Challenges identified include a lack of institutional capacity and communication problems between the various stakeholders involved in purchasing health services. |
| O. Onwujekwe et al [35] | 2019/Qualitative study/Nigeria | ***Health financing mechanisms:***  Funding sources include the government, individuals and households, as well as contributions from policyholders of the Fund for Social Health Insurance Program (FSSHIP) and community health insurance, and from donors. These funds are then consolidated in a fragmented way by the government, with FSSHIP using Health Maintenance Organizations (HMOs) to purchase health services, community health insurance acting directly or through third parties, and donors adopting various purchasing models depending on their assessment of financial risk.  ***Challenges:***  Nigeria faces significant health financing challenges, including inefficient funding, inequitable distribution of resources, poor quality of care, the financial impact on households and the sustainability of current financing mechanisms. |
| K. Damrongplasit et G. Melnick [21] | 2015/Quantitative study/Thailand | ***Health financing mechanisms:***  healthcare expenditure is financed by public (three quarters) and private (one quarter) sources. The Civil Servant Medical Benefit Scheme (CSMBS), for civil servants, uses fee-for-service payment and Diagnosis-Related Groups (DRGs), while the Social Security Scheme (SSS), for employees in the formal sector, uses fixed capitation payments. The Universal Coverage Scheme (UCS), for the informal sector and the uninsured, pays contracted providers by capitation.  ***Challenges:***  the continuing increase in expenditure on the civil servants' scheme and the under-funding of the CSU  ***key lessons learned:***  Thailand has succeeded in completing the CSU thanks to its financing system based on public sources, a low rate of direct payments, and extended service coverage. |
| E. Richardson et N. Berdzuli [29] | 2017/Review/Georgia | ***Health financing mechanisms:***  Health financing relies mainly on direct household spending, government revenues and private health insurance programs. Public resources are pooled primarily through the Social Services Agency (SSA), as well as private health insurance companies. The transition to a contractual model between purchasers and providers, with fee-for-service as the current method.  ***Challenges:***  high out-of-pocket payment rates and healthcare quality are persistent challenges.  ***key lessons learned:***  UHCP is financially supported by the state budget, which has led to an increase in the use of health services and a decrease in direct payments. The SSA, as the main purchaser, has significantly reduced the fragmentation of the system and improved its efficiency. |
| B. S. C. Uzochukwu ei al [16] | 2015/Systematic review/Nigeria | ***Health financing mechanisms:***  The main source of funding for healthcare in Nigeria is direct payment by households(69%), and the government also funds healthcare through tax revenues, which are divided between the federal, state and local governments. Donor funding also contributes to financing. The pooling of resources to finance healthcare in Nigeria is mainly through health insurance.  ***Challenges:***  In the Nigerian context, the financing structure faces a number of problems, first and foremost the inadequate political commitment to health, the problems of NHIS governance, and the dominance of direct payments. |
| M. Obadha et al [49] | 2019/A cross‐sectional qualitative approach/ Kenya | ***Health financing mechanisms:***  The Kenyan healthcare system has three purchasing modalities: the public integrated model, where the national Ministry of Health pays tertiary public providers via global budgets, county governments manage primary and secondary public providers to which they allocate individual budgets( per item) to providers, the public contract model used by the NHIF which pays according to capitation and FFS, and finally, the private purchasing model where private insurers use FFS for public, private and faith-based providers.  ***Challenges:***  the inadequacy of NHIF capitation rates per individual, delays in receiving payments from NHIF and private insurers, and the lack of autonomy of public providers in the use of NHIF funds. |
| K. Zhu et al [54] | 2017/Quantitative study/China | ***Health financing mechanisms:***  The challenges include the low levels of funding for the Urban Resident Basic Medical Insurance (URBMI) and the New Rural Cooperative Medical Scheme (NCMS) in China. There is also an issue of inequity in individual contributions between urban and rural residents. Additionally, there is a lack of institutional arrangements for fiscal responsibility for contributions to the Urban and rural Residents Basic Medical Insurance (URRBMI) funds. |
| S. C. Ifeagwu et al [13] | 2021/Systematic review/ Sub-Saharan Africa | ***Health financing mechanisms:***  Health financing mechanisms generally used in the region include national health insurance (NHI), community-based health insurance (CBHI), tax-based financing, external financing, other forms of health insurance and innovative approaches such as increasing public spending on health and applying taxes on products such as alcohol and tobacco.  ***Challenges:***  the dependence of the majority of countries in the region on regressive forms of financing such as out-of-pocket spending, as well as dependence on donor funding, are challenges faced by most countries in the region. another persistent challenge is access to quality health services for vulnerable populations. |
| C. Atim et al [18] | 2021/Qualitative case studies/Ghana, India, Indonesia, Kenya, and South Africa | ***Health financing mechanisms:***  Different countries employ varied models for financing and purchasing healthcare services. In Ghana, the National Health Insurance Scheme (NHIS) is funded by public taxes and premiums. India's Ayushman Bharat program is state-funded, with implementation managed at the state level, leading to fragmentation. Indonesia's Jaminan Kesehatan Nasional (JKN) is a universal scheme funded by payroll taxes. Kenya's National Hospital Insurance Fund (NHIF) is financed by compulsory premiums for the formal sector and voluntary contributions for the informal sector. South Africa relies on voluntary health insurance, with the public sector utilizing progressive budgets and the private sector employing a fee-for-service (FFS) system.  ***Challenges:***  Inadequate public funding of healthcare remains a major obstacle in most of the countries studied, as do insufficient service delivery capacity and problems with the quality of care. |
| N. Hatam et al [24] | 2021/Mixt methods (quantitative-qualitative)/  Iran, Turkey, Thailand and China | ***Health financing mechanisms:***  In the countries studied, healthcare services are financed from a variety of sources, including taxes, public resources, social health insurance, out-of-pocket payments and private sources. In Turkey and Thailand, the functions of purchaser and supplier are distinct, with government agencies and private insurers purchasing healthcare services. In China, government agencies are limited in their ability to negotiate tariffs and control the quality of services. Different payment methods are used in the countries studied, such as per capita, DRG, fee-for-service, salary and performance-based payments. In Thailand, ambulatory services are paid per head, while preventive services include performance-based payments.  ***Challenges:***  A significant proportion of healthcare expenditure comes from the private sector(Iran). The challenges associated with pooling resources are obvious, notably the consolidation of insurance funds, the multiplicity of insurance companies, disparities in premiums and payment commitments, the lack of information structures, and the presence of an informal economic sector. As far as purchasing mechanisms are concerned, fee-for-service payment has a significant impact on service quality and provider motivation.  ***key lessons learned:***  Countries that have made progress in achieving CSU objectives have done so by increasing the share of public sector funding, reducing direct payments, reducing risk pooling funds, making strategic purchases and using payment methods with a specified limit to reduce costs. |
| L. Doshmangir et al [41] | 2021/Qualitative study(Retrospective policy analysis)/ Iran | ***Challenges:***  the gap between private and public sector tariffs has widened considerably, reducing access to services and increasing costs in the private sector, fragmentation of health insurance pooling, high financial burden jeopardizing the financial viability of the scheme covering the poor, rural residents and the self-employed. |
| E. Barasa et al [17] | 2018/Qualitative study/ Kenya | ***Health financing mechanisms:***  The country has a mixed system of healthcare funding, financed by public revenues (taxes and donors), health insurance contributions and OOP. Purchasing of healthcare services is carried out through (1) integrated purchasing between public facilities by national and county governments (2) the NHIF, which contracts with public and private healthcare facilities in Kenya; and (3) private health insurance companies which contract with private healthcare facilities.  ***Challenges:***  Unequal access to health services, weak institutional and technical capacity, and the long-term financial viability of the NHIF. |
| M. Á. González Block et al [32] | 2020/Review/Mexico | ***Health financing mechanisms:***  Revenue sources in the Mexican financing system include public sources, which account for 53.8% of total healthcare expenditure, and a significant proportion (46.2%) from private sources, of which the OOP is the main source of funding at 41.3%. Limited pooling of funds within each social insurance institution and government program. Social insurance institutions integrate the purchase and provision of healthcare services; provider payment models vary from payer to payer and provider to provider. The dominant payment model is the historical budget, although other payment methods are used, such as capitation and fee-for-service.  ***Challenges:***  OOP remains the main problem in Mexico's financing system. |
| G. Lagomarsino et al [33] | 2012/Qualitative exploratory study /Ghana, Indonesia, Philippines, Rwanda, Vietnam, India, Kenya, Mali and Nigeria/ | ***Health financing mechanisms:***  Several strategies are used by different countries to raise revenue, including general taxes, earmarked taxes, payroll deductions and household contributions. Only in Kenya (36%), Mali (27%) and Rwanda (47%) do donor expenditures account for more than a quarter of funding. Some countries adopt a progressive approach, starting with different risk pools for different target populations, while others have opted for a single risk pool to cover the entire population, whether rich or poor, formal or informal. In terms of purchasing health care services, the nine countries we have selected appear to be moving towards demand-driven purchasing for at least certain types of care and certain populations. Several payment methods have been adopted by these countries, such as capitation, case-based payments, as in Ghana and Vietnam, and performance-based payments, as in Rwanda.  ***Challenges:***  The importance of the informal economy and passive purchasing of healthcare services. Moreover, in most of the selected countries, quality of care remains a major challenge.  ***key lessons learned:***  Social health insurance is essential to protect individuals financially against the risks of illness. |
| C. Li et al [20] | 2011/Evaluative document review/  China | ***Health financing mechanisms:***  The Urban Employee Basic Medical Insurance (UEBMI) in China is funded jointly by employers and employees through payroll taxes, while the New Rural Cooperative Medical Scheme (NRCMS) receives contributions from the central government, local governments, and individuals, with increasing individual contributions over time. The Urban Resident Basic Medical Insurance (URBMI) relies primarily on household or individual contributions, supplemented by public subsidies. Pooling of insurance funds occurs at the municipal level for UEBMI and URBMI and at the county level for NRCMS. Despite varying benefit packages, all three schemes predominantly use fee-for-service payment for healthcare providers, although alternative methods like capitation, Diagnosis Related Groups (DRG), and global budgets are also employed.  ***Challenges:***  There are still several obstacles to overcome because of insufficient financial protection, fragmented risk pooling, inefficient fee-for-service system, regional disparities in funding and reimbursement rates, and inequalities in access to health care. |
| D. McIntyre et al [31] | 2013  /Qualitative analysis/ Costa Rica, Georgia, India, Malawi, Nigeria, Tanzania and Thailand. | ***Challenges:***  The fragmentation of funds leads to differences in benefits between schemes, and the limited capacity of the purchasing organization.  ***key lessons learned:***  Public financing has played a crucial role in advancing countries towards universal coverage. Moreover, countries offering comprehensive services have been able to reduce the rate of out-of-pocket (OOP) expenditures in total health expenditures (THE) and decrease catastrophic expenses. Additionally, concentrating purchasing power under a single buyer, the separation between the purchaser and provider, and the creation of an autonomous body responsible for the purchase of health services are key factors in achieving universal health coverage goals. |
| N. Devadasan et al [22] | 2013/Mixed methods (qualitative-quantitative)/ India | ***Health financing mechanisms:***  India’s financing system relies primarily on OOP at 72%. For the RSBY scheme, it is funded by the Indian government with a symbolic contribution from registrants. The RSBY operates through private insurance companies. Hospitals pay the insurance companies using Diagnosis-Related Group (DRG) packages.  ***Challenges:***  The high rate of out-of-pocket payments pushes many Indian people into poverty. |
| L. Popovich et al [27] | 2011/Review/Russian Federation | ***Health financing mechanisms:***  Russia have a mixte funding sources for healthcare, including mandatory contribution, direct payments, and other sources like private health insurance and NGOs. There are two main types of pools for prepaid funds: Mandatory Health Insurance (MHI) (through its federal and territorial funds) and budgets at different levels. Funds from the Mandatory Health Insurance (MHI), collected through payroll contributions, are transferred to insurance companies. These companies contract with healthcare providers for service delivery and used case-based payments. For regional or local budgets, the relationships between the purchaser and the suppliers are more integrated using historical budgetary processes.  ***Challenges:***  OOP payment burden poor households, while regional disparities in public funding raise concerns about equity. |
| R. Pokharel et P. R. Silwal [26] | 2018/Review/ Nepal | ***Health financing mechanisms:***  The main sources of funding for the social health insurance in Nepal include household contributions (formal/informal sectors) and funding from various levels of government. The SHI system operates as a single fund at the central level, aiming to gradually consolidate existing fragmented schemes under the Ministry of Health into a single fund. It offers a set of benefits, utilizing different payment mechanisms based on the types of services provided to pay providers. Outpatient and emergency services are paid per case, DRGs for hospitalization, and fee-for-service for certain services.  ***Challenges:***  The high contribution of personal expenses leads to high catastrophic expenses, the collection of contributions from the informal sector is administratively expensive, and the viability of the health insurance system are major challenges. |
| S. Kwon et L. Keo [25] | 2019/Qualitative case study/ Cambodia | ***Health financing mechanisms:***  The Health Equity Fund (HEF), jointly funded by the government and donors, and the National Social Security Fund (NSSF) in Cambodia, primarily supported by contributions. The HEF purchases services from public providers according to standardized guidelines, with payment processing managed by the Payment Certification Agency (PCA). while the NSSF purchases standardized service packages, both from public and private providers, using a case-based payment system.  ***Challenges:***  In general, Cambodia faces challenges in providing healthcare, including ensuring quality of care, governance issues, and expanding coverage to reach all vulnerable populations. The reliance on OOP remains a significant concern in Cambodia's healthcare system. |
| U. Ezenwaka et al [51] | 2022/Qualitative, descriptive case-study/ Nigeria | ***Health financing mechanisms:***  In Nigeria, the federal government serves as the primary purchaser of healthcare services, operating through the Federal Ministry of Health and the National Primary Health Care Development Agency. Contractual arrangements with providers are informal for public establishments and selective for private ones. The National Health Insurance Scheme (NHIS) also engages in selective contracting with providers through Health Maintenance Organizations (HMOs). Payment to providers is based on budget allocations for the federal government and utilizes capitation and fee-for-service payments for the NHIS.  ***Challenges:***  Several challenges hinder the effective implementation of strategic purchasing of healthcare products in Nigeria. Firstly, a very limited proportion of health financing worsened by high fragmentation of funding. Secondly, non-compliance with contractual agreements. Thirdly, delays in approval and payment of reimbursement claims to providers. Fourthly, fee-for-service and global budget payment models do not incentivize provider performance. Fifthly, weak or nonexistent oversight of healthcare purchasing activities. Lastly, underutilization of data from information systems for purchasing decisions. |
| E. Amporfu et al [71] | 2022/Qualitative descriptive study/ Ghana | ***Health financing mechanisms:***  In Ghana, the National Health Insurance Scheme (NHIS) relies mainly on designated taxes, such as value-added taxes (which account for 72% of NHIS revenue), as well as workers' social security contributions (20%) and non-exempt members' contributions (which vary from 2% to 5%). The NHIS mainly covers curative care in various healthcare facilities. With the exception of public facilities, other healthcare providers enter into selective contracts with the National Health Insurance Authority (NHIA), using various payment methods based on Ghana-Diagnosis Related Groupings (G-DRG) for most outpatient and inpatient services, as well as fee-for-service for drugs and non-grouped services. Performance monitoring includes annual accreditation, ad hoc clinical audits, customer surveys and feedback forms.  ***Challenges:***  Insufficient public funds, delayed payment of funds to NHIA and ongoing budget deficits, inefficient payment of providers (G-DRGs), billing of unapproved and unauthorized fees to members for certain services.  ***key lessons learned:***  The NHIS governance is strong institutionally, with the NHIA effectively managing purchasing. The service package provided aligns with public health strategies and the country's epidemiological profile. |
| W. Patcharanarumol et al [56] | 2018/A mixed method (Qualitative/Quantitative)/  Thailand | ***Health financing mechanisms:***  The Comptroller General's Department (CGD) oversees the Civil Servant Medical Benefit Scheme (CSMBS) as part of civil servant social protection. Their healthcare purchasing is passive. Fee-for-service outpatient care has led to cost escalation, surpassing their budget. In contrast, the National Health Security Office (NHSO) manages purchasing for the Universal Coverage Scheme (UCS), implementing strategic purchasing actions like closed-provider payments and promoting primary healthcare gatekeeping.  ***Challenges:***  CSMBS suffers from high costs, excessive use of non-essential drugs and low quality of service/ CGD lacks commitment and technical capacity to introduce effective reforms.  ***key lessons learned:***  Strategic purchasing can improve healthcare system efficiency by relying on primary care as gatekeepers, using closed budgets with appropriate payment methods for providers, implementing the National List of Essential Medicines (NLEM) effectively. These efforts are supported by key elements such as legal frameworks, institutional arrangements by strengthening the capacity of purchasers and suppliers |
| E. Etiaba et al [52] | 2018/Qualitative study/Nigeria | ***Health financing mechanisms:***  The National Health Insurance Scheme (NHIS) procures tertiary care services directly from state and federal tertiary care providers, while primary and secondary care services are procured through Health Maintenance Organizations (HMOs). Benefit packages are standardized for all enrollees and determined by the NHIS, which includes federal civil servants and the organized private sector (formal private sector organizations with more than 10 employees). Various payment methods are utilized based on the service type, including capitation and fee-for-service (FFS).  ***Challenges:***  Payment delays, dissatisfaction with payment rates and lack of transparency in the decision-making process. |
| A. P. Fenny et al [19] | 2021/Systematic review/ Ghana, Rwanda, Tanzania, Kenya and Ethiopia | ***Health financing mechanisms:***  Except for Ghana, which has implemented a national health insurance scheme for its entire population, Tanzania, Kenya, and Rwanda have each established fragmented health insurance schemes offering coverage to their populations based on their socioeconomic status. These countries finance their health reforms through a combination of sources such as donors, the public sector, households, and other private sector actors.  ***Challenges:***  Including the informal sector and guaranteeing compulsory membership of social health insurance schemes are recurring challenges in these countries. Dependence on public and external funding poses risks for the sustainability of health insurance schemes. In addition, the fragmentation of risk pools can prevent the implementation of effective cross-subsidization mechanisms.  ***key lessons learned:***  Financing systems that rely less on direct expenditure, as seen in Rwanda, demonstrate a higher level of risk mutualization. |
| S. M. Umuhoza et al [43] | 2022/Qualitative study/Rwanda | ***Health financing mechanisms:***  The Ministry of Health specifies the covered services for CBHI and RSSB schemes, granting members access to comprehensive care from public and private healthcare facilities. Both schemes establish contractual arrangements with providers, including automatic engagement for public facilities and selective contracts for certain private providers. Payments to providers are typically fee-for-service, with performance-based contracts under the PBF system. Various performance monitoring mechanisms, such as field visits, inspections, audits, and regular evaluations, ensure service quality.  ***Challenges:***  Weak governance mechanisms for the purchasing of healthcare services, manifested by redundant functions between the institutions responsible for this financing function. There is no clear methodology for defining and regularly updating service packages. In addition, the variability of service packages can contribute to inequity, while contractual agreements with public institutions limit the scope for strategic purchasing. non-application of performance monitoring procedures and lack of interoperability between information systems. |
| C. Mbachu et al [53] | 2021/A mixed methods (Qualitative/Quantitative)/ Nigeria | ***Health financing mechanisms:***  In Nigeria, the General Taxi Revenue (GTR) scheme involves contracts between the State Ministry of Health (SMOH) and public healthcare providers, with no selective arrangements for private providers. Payment is based on a fixed salary. The Social Health Insurance Scheme (SSHIS) establishes selective contracts with providers through the State Health Insurance Agency (IMSHIA), implementing performance-based contracts. SSHIS offers a tailored service package covering various healthcare services, remunerating providers through capitation or fee-for-service methods and by per diem for hospitalization.  ***Challenges:***  Weak governance in the GTR system, lacking separation between buyer and supplier, no rules for selective contracting, and limited performance-based management. Health financing faces constraints with low public spending and high out-of-pocket payment. Technical capacity is lacking, particularly in electronic claims management. |
| Q. N. Le, L. Blizzard et al [23] | 2020/Desk review/Vietnam | ***Health financing mechanisms:***  The social health insurance scheme (SHI) is financed by fixed insurance premiums and public subsidies for vulnerable categories. It operates on the principle of risk pooling, with funds pooled in a single fund managed by the Viet Nam Social Security (VSS). The agency plays an essential role in purchasing healthcare services from providers. The benefit package includes a wide range of healthcare services, and VSS finances these services through fee-for-service payment, capitation, and payment by diagnostic group (DRG).  ***Challenges:***  Vietnam lacks an effective mechanism for identifying the uninsured, particularly those in the informal sector. Additionally, the country lacks specific criteria for determining which benefits should be included in the Social Health Insurance (SHI) benefit package.  ***key lessons learned:***  Social health insurance accelerated progress towards universal public health by merging disparate provincial funds into a single, financially sound fund, thus promoting risk-sharing among health insurance members. And, Subsidies played a key role in extending health insurance coverage. |
| N. Milevska Kostova et al [28] | 2017/Review/North Macedonia | ***Health financing mechanisms:***  The healthcare system in North Macedonia is financed mainly by compulsory insurance contributions, general taxation and direct expenditure. It is based on a health insurance model that centralizes funds within the Health Insurance Fund (HIF). The Ministry of Health also negotiates its annual budget with the Ministry of Finance. The HIF manages the purchasing process for healthcare services by concluding contracts, using different payment methods for providers, such as DRG, conditional budgets, fee-for-service, capitation and payment by performance (P4P).  ***Challenges:***  OOP payment and regional disparities in access to healthcare  ***key lessons learned:***  The health insurance system in North Macedonia is based on a single-buyer model, which gives us negotiating power with providers, avoiding fragmentation of resources and minimizing administrative costs. |
| M. Tatar et al [36] | 2011/Review/Turkey | ***Health financing mechanisms:***  Turkey finances healthcare services from a variety of sources, mainly social health insurance contributions, followed by government sources and direct payments. The public purchasers of the Turkish healthcare system are ISS and the government. Healthcare services in Turkey are purchased from both public and private providers, with varying payment terms. Public hospitals receive annual budgets from the government and are also paid by the Social Security Institution (SSI) and patients on a fee-for-service basis. Individual providers receive a fixed salary and performance-based incentives. For public-sector providers, no contractual agreement is required with the SSI, while for the private sector, contracts are negotiated directly.  ***Challenges:***  Government subsidies offset the financial deficits of Turkey's health insurance schemes. Budget overruns are recurrent, and there is no mechanism for assessing the quality of services. There are also concerns about the impact of provider-induced demand.  ***key lessons learned:***  Public funding plays a crucial role in reducing OOP payments in Turkey. |
| H. Yu [42] | 2015/ Qualitative analysis/ China | ***Health financing mechanisms:***  China has succeeded in achieving universal health coverage thanks to these three pooling mechanisms. notably, the New Rural Cooperative Medical Scheme (NRCMS), launched in 2003 in rural areas, Urban Resident Basic Medical Insurance (URBMI), launched in 2007 to target the unemployed, children, students and disabled in urban areas, and Urban Employee Basic Medical Insurance (UEBMI), launched in 1998 as an employment-based insurance program.  ***Challenges:***  the high level of out-of-pocket payments constitute a major challenge, as do the significant disparities between premiums and benefits under the various insurance programs. In addition, the low quality of healthcare and the rapid growth in healthcare costs are major concerns. |
| J. Chuma et V. Okungu [40] | 2011/Mixed Methods(qualitative-quantitative)/ Kenya | ***Health financing mechanisms:***  Au Kenya, les fonds sont collectés à partir de diverses sources de financement, notamment les paiements directs restent la principale source de financement de la santé au Kenya, Les allocations gouvernementals, les donateurs , et l'assurance maladie. Les principales formes de mise en commun(seulement 4 % des fonds sont mise en commun par l'assurance maladie) comprennent le National Hospital Insurance Fund (NHIF)( le plus grand pool de risque du pays), l'assurance maladie privée, les Community-Based Health Insurance (CBHI) et le financement par les donateurs par le biais de l'aide budgétaire générale. Au Kenya, les services de santé sont achetés par divers organismes, notamment le Ministère des Services Médicaux et le Ministère de la Santé Publique et de l'Assainissement pour les établissements publics, ainsi que les collectivités locales, le NHIF, les CBHI, les assurances maladie privées et les employeurs. Les prestataires publics sont rémunérés par un budget général et un salaire, tandis que le NHIF paie les prestataires accrédités avec un taux journalier forfaitaire. Les compagnies d'assurance privées paient les hôpitaux agréés selon des honoraires par cas ou à l'acte.  ***Challenges:***  Out-of-pocket payments are still the main source of healthcare funding in Kenya, and the pooling of healthcare resources is limited, due to the fragrance of the funding system, and the poor quality of the services offered. |
| Z. G. Ökem et M. Çakar [39] | 2015/Systematic review/Turkey | ***Health financing mechanisms:***  SSI is becoming the main purchaser of healthcare services in Turkey by consolidating health insurance schemes and concluding contracts with healthcare providers. Healthcare services are provided with a comprehensive, harmonized package of benefits for all members of the compulsory health insurance scheme. Payments for healthcare services include salaries and performance-related payments, capitation, as well as budget ceilings.  ***Challenges:***  Long-term financial sustainability remains a challenge, Performance-based payments, can lead to inefficiencies, regional inequalities limits access to healthcare and creates inequalities in service delivery and Challenges related to the quality of healthcare services.  ***key lessons learned:***  The consolidation of health insurance schemes within ISS has helped to control expenditure. The implementation of reference prices, reimbursement mechanisms and global budget ceilings has kept costs down. Performance-based payments have stimulated service delivery and improved hospital efficiency. |

**Supplementary Material 4.** Quality Check List.

***Quality Check List for Quantitative studies (JBI tool)***

| **Articles/Questions** | **1. Were the criteria for inclusion in the sample clearly defined?** | **2. Were the study subjects and the setting described in detail?** | **3. Was the exposure measured in a valid and reliable way?** | **4. Were objective, standard criteria used for measurement of the condition?** | **5. Were confounding factors identified?** | **6. Were strategies to deal with confounding factors stated?** | **7. Were the outcomes measured in a valid and reliable way?** | **8. Was appropriate statistical analysis used?** | **Quality** |
| --- | --- | --- | --- | --- | --- | --- | --- | --- | --- |
| **Funding, Coverage, and Access Under Thailand’s Universal Health Insurance Program: An Update After Ten Years** | YES | YES | Can’t Tell | NO | YES | YES | YES | YES | **Medium** |
| **Health financing and integration of urban and rural residents' basic medical insurance systems in China.** | NO | YES | YES | YES | YES | NO | YES | Can’t Tell | **Medium** |

***Quality Check List for Qualitative studies: (CASP) checklist***

| **Articles/Questions** | **1.Was there a clear statement of the aims of theresearch?** | **2. Is a qualitative methodology appropriate?** | **3. Was the research design appropriate to address the aims of the research?** | **4. Was the recruitment strategy appropriate to the aims of the research?** | **5. Was the data collected in a way that addressed the research issue?** |  | **6. Has the relationship between researcher participants been adequately considered?** | **7. Have ethical issues been taken into consideration** | **8. Was the data analysis sufficiently rigorous?** | **9. Is there a clear statement of findings?** | **10. How valuable is the research** | **Quality** |
| --- | --- | --- | --- | --- | --- | --- | --- | --- | --- | --- | --- | --- |
| **A critical analysis of health care purchasing arrangements in Kenya: A case study of the county departments of health** | YES | YES | YES | YES | YES |  | CAN'T TELL | YES | YES | YES | YES | **High** |
| **A critical analysis of purchasing arrangements in Kenya: The case of micro health insurance** | YES | YES | YES | YES | YES |  | YES | YES | YES | YES | YES | **High** |
| **A critical analysis of purchasing arrangements in Kenya: The case of the national hospital insurance fund** | YES | YES | YES | YES | YES |  | CAN'T TELL | YES | YES | YES | YES | **High** |
| **Examining purchasing reforms towards universal health coverage by the National Hospital Insurance Fund in Kenya** | YES | YES | YES | YES | YES |  | YES | YES | YES | YES | YES | **High** |
| **Health care purchasing in Kenya: Experiences of health care providers with capitation and fee-for-service provider payment mechanisms** | YES | YES | YES | YES | YES |  | No | YES | YES | YES | YES | **High** |
| **Kenya national hospital insurance fund reforms: Implications and lessons for universal health coverage** | YES | YES | YES | No | YES |  | No | CAN'T TELL | YES | YES | YES | **Medium** |
| **A qualitative study of the difficulties in reaching sustainable universal health insurance coverage in Iran** | YES | YES | YES | YES | YES |  | YES | YES | YES | YES | YES | **High** |
| **Iran health insurance system in transition: equity concerns and steps to achieve universal health coverage** | YES | YES | YES | YES | YES |  | No | YES | YES | YES | YES | **High** |
| **Achieving universal health coverage goals in Thailand: The vital role of strategic purchasing** | YES | No | YES | No | YES |  | No | CAN'T TELL | YES | YES | YES | **Medium** |
| **Exploring effectiveness of different health financing mechanisms in Nigeria; what needs to change and how can it happen?** | YES | YES | YES | YES | YES |  | YES | YES | YES | YES | YES | **High** |
| **Strategic purchasing for universal health coverage: examining the purchaser–provider relationship within a social health insurance scheme in Nigeria** | YES | YES | YES | YES | YES |  | CAN'T TELL | YES | YES | YES | YES | **High** |
| **Strategic Health Purchasing in Nigeria: Investigating Governance and Institutional Capacities within Federal Tax-Funded Health Schemes and the Formal Sector Social Health Insurance Programme** | YES | YES | YES | YES | YES |  | YES | YES | YES | YES | YES | **High** |
| **Examining healthcare purchasing arrangements for strategic purchasing in Nigeria: a case study of the Imo state healthcare system** | YES | YES | YES | YES | YES |  | YES | YES | YES | YES | YES | **High** |
| **Strategic Health Purchasing Progress Mapping: A Spotlight on Ghana's National Health Insurance Scheme.** | YES | YES | YES | YES | YES |  | No | YES | YES | YES | YES | **High** |
| **Assessment of Strategic Healthcare Purchasing Arrangements and Functions Towards Universal Coverage in Tanzania** | YES | YES | YES | YES | YES |  | No | YES | YES | YES | YES | **High** |
| **Strengths and Weaknesses of Strategic Health Purchasing for Universal Health Coverage in Rwanda.** | YES | YES | YES | YES | YES |  | YES | YES | YES | YES | YES | **High** |
| **Assessment of systems for paying health care providers in Vietnam: Implications for equity, efficiency and expanding effective health coverage** | YES | YES | YES | YES | YES |  | CAN'T TELL | YES | YES | YES | YES | **High** |
| **Universal health insurance coverage for 1.3 billion people: What accounts for China's success?** | YES | YES | YES | CAN'T TELL | YES |  | CAN'T TELL | CAN'T TELL | CAN'T TELL | YES | YES | **Medium** |
| **Moving towards universal health coverage: Health insurance reforms in nine developing countries in Africa and Asia** | YES | YES | YES | CAN'T TELL | YES |  | CAN'T TELL | CAN'T TELL | YES | YES | YES | **Medium** |
| **Promoting universal financial protection: evidence from seven low- and middle-income countries on factors facilitating or hindering progress** | YES | YES | YES | CAN'T TELL | YES |  | CAN'T TELL | CAN'T TELL | YES | YES | YES | **Medium** |
| **Social health protection in Cambodia: Challenges of policy design and implementation** | YES | YES | YES | CAN'T TELL | CAN'T TELL |  | CAN'T TELL | CAN'T TELL | CAN'T TELL | YES | YES | **Low** |
| **Achieving universal health coverage goals in Thailand: The vital role of strategic purchasing** | YES | CAN'T TELL | CAN'T TELL | CAN'T TELL | CAN'T TELL |  | No | No | CAN'T TELL | YES | YES | **Low** |
| **cette étude explore la mise en œuvre par les PRFM de mécanismes de financement de la santé spécifiques au contexte et visant à atteindre la CSU : Le régime national d'assurance maladie au Ghana comme cas test** | YES | CAN'T TELL | YES | CAN'T TELL | YES |  | No | No | CAN'T TELL | YES | YES | **Low** |
| **Health financing reforms for Universal Health Coverage in five emerging economies** | YES | CAN'T TELL | YES | CAN'T TELL | YES |  | No | No | CAN'T TELL | YES | YES | **Low** |

***Quality Check List for Mixed studies: (MMAT) checklist***

| **Articles/Questions** | **1. Is there an adequate rationale for using a mixed methods design to address the research question?** | **2. Are the different components of the study effectively integrated to answer the research question?** | **3. Are the outputs of the integration of qualitative and quantitative components adequately interpreted?** | **4. Are divergences and inconsistencies between quantitative and qualitative results adequately addressed?** | **5. Do the different components of the study adhere to the quality criteria of each tradition of the methods involved?** | **Quality** |
| --- | --- | --- | --- | --- | --- | --- |
| **Healthcare System Functions in Iran and Successful Developing Countries Regarding Access to Universal Health Coverage: A Comparative Study** | Yes | Yes | Yes | Yes | Yes | **High** |
| **Promoting universal financial protection: evidence from the Rashtriya Swasthya Bima Yojana (RSBY) in Gujarat, India** | Yes | Yes | Yes | Yes | Yes | **High** |
| **Strategic purchasing and health system efficiency: A comparison of two financing schemes in Thailand.** | Yes | Yes | Yes | Yes | Yes | **High** |
| **Supporting strategic health purchasing: a case study of annual health budgets from general tax revenue and social health insurance in Abia state, Nigeria** | Yes | Yes | Yes | Yes | Yes | **High** |
| **Viewing the Kenyan health system through an equity lens: implications for universal coverage** | Yes | Yes | Yes | No | Yes | **Medium** |

***Quality Check List for Systematic reviews: (CASP) checklist***

| **Articles/Questions** | **1. Did the review address a clearly focused question?** | **2. Did the authors look for the right type of papers?** | **3. Do you think all the important, relevant studies were included?** | **4. Did the review’s authors do enough to assess quality of the included studies?** | **5. If the results of the review have been combined, was it reasonable to do so?** | **6. What are the overall results of the review?** | **7. How precise are the results?** | **8. Can the results be applied to the local population?** | **9. Were all important outcomes considered?** | **10. Are the benefits worth the harms and costs?** | **Quality** |
| --- | --- | --- | --- | --- | --- | --- | --- | --- | --- | --- | --- |
| **A systematic review of the health-financing mechanisms in the Association of Southeast Asian Nations countries and the People’s Republic of China: Lessons for the move towards universal health coverage** | YES | YES | YES | YES | Can’t Tell | YES | NO | NO | NO | NO | **Low** |
| **Health care financing in Nigeria: Implications for achieving universal health coverage** | YES | YES | Can’t Tell | Can’t Tell | Can’t Tell | YES | Can’t Tell | YES | Can’t Tell | Can’t Tell | **Low** |
| **Health financing for universal health coverage in Sub-Saharan Africa: a systematic review** | YES | YES | Can’t Tell | YES | YES | YES | Can’t Tell | YES | YES | Can’t Tell | **Medium** |
| **Strategies for financing social health insurance schemes for providing universal health care: a comparative analysis of five countries** | YES | YES | YES | NO | Can’t Tell | YES | Can’t Tell | YES | YES | NO | **Medium** |
| **What have health care reforms achieved in Turkey? An appraisal of the “Health Transformation Programme”** | YES | YES | Can’t Tell | NO | Can’t Tell | YES | Can’t Tell | Can’t Tell | YES | Can’t Tell | **Low** |
| **A systematic review of the health-financing mechanisms in the Association of Southeast Asian Nations countries and the People’s Republic of China: Lessons for the move towards universal health coverage** | YES | YES | Can’t Tell | YES | YES | YES | Can’t Tell | Can’t Tell | YES | NO | **Medium** |
